# FGFR2-triggered autophagy and activation of Nrf-2 reduce breast cancer cell response to anti-ER drugs

**DOI:** 10.1186/s11658-024-00586-6

**Published:** 2024-05-14

**Authors:** Monika Gorska-Arcisz, Marta Popeda, Marcin Braun, Dominika Piasecka, Joanna I. Nowak, Kamila Kitowska, Grzegorz Stasilojc, Marcin Okroj, Hanna M. Romanska, Rafal Sadej

**Affiliations:** 1 Laboratory of Enzymology and Molecular Oncology, Intercollegiate Faculty of Biotechnology, University of Gdansk and Medical University of Gdansk, Debinki 1, 80-211 Gdansk, Poland; 2 Department of Pathomorphology, Medical University of Gdansk, Gdansk, Poland; 3 Department of Pathology, Chair of Oncology, Medical University of Lodz, Pomorska 251, 92-213 Lodz, Poland; 4 Department of Histology, Medical University of Gdansk, Gdansk, Poland; 5 Department of Cell Biology and Immunology, Intercollegiate Faculty of Biotechnology, University of Gdansk and Medical University of Gdansk, Gdansk, Poland

**Keywords:** FGFR2, Autophagy, p62, Keap1, Nrf-2, Luminal breast cancer

## Abstract

**Background:**

Genetic abnormalities in the FGFR signalling occur in 40% of breast cancer (BCa) patients resistant to anti-ER therapy, which emphasizes the potential of FGFR-targeting strategies. Recent findings indicate that not only mutated FGFR is a driver of tumour progression but co-mutational landscapes and other markers should be also investigated. Autophagy has been recognized as one of the major mechanisms underlying the role of tumour microenvironment in promotion of cancer cell survival, and resistance to anti-ER drugs. The selective autophagy receptor p62/SQSTM1 promotes Nrf-2 activation by Keap1/Nrf-2 complex dissociation. Herein, we have analysed whether the negative effect of FGFR2 on BCa cell response to anti-ER treatment involves the autophagy process and/or p62/Keap1/Nrf-2 axis.

**Methods:**

The activity of autophagy in ER-positive MCF7 and T47D BCa cell lines was determined by analysis of expression level of autophagy markers (p62 and LC3B) and monitoring of autophagosomes’ maturation. Western blot, qPCR and proximity ligation assay were used to determine the Keap1/Nrf-2 interaction and Nrf-2 activation. Analysis of 3D cell growth in Matrigel® was used to assess BCa cell response to applied treatments. In silico gene expression analysis was performed to determine FGFR2/Nrf-2 prognostic value.

**Results:**

We have found that FGFR2 signalling induced autophagy in AMPKα/ULK1-dependent manner. FGFR2 activity promoted dissociation of Keap1/Nrf-2 complex and activation of Nrf-2. Both, FGFR2-dependent autophagy and activation of Nrf-2 were found to counteract the effect of anti-ER drugs on BCa cell growth. Moreover, in silico analysis showed that high expression of *NFE2L2* (gene encoding Nrf-2) combined with high *FGFR2* expression was associated with poor relapse-free survival (RFS) of ER+ BCa patients.

**Conclusions:**

This study revealed the unknown role of FGFR2 signalling in activation of autophagy and regulation of the p62/Keap1/Nrf-2 interdependence, which has a negative impact on the response of ER+ BCa cells to anti-ER therapies. The data from in silico analyses suggest that expression of Nrf-2 could act as a marker indicating potential benefits of implementation of anti-FGFR therapy in patients with ER+ BCa, in particular, when used in combination with anti-ER drugs.

**Graphical Abstract:**

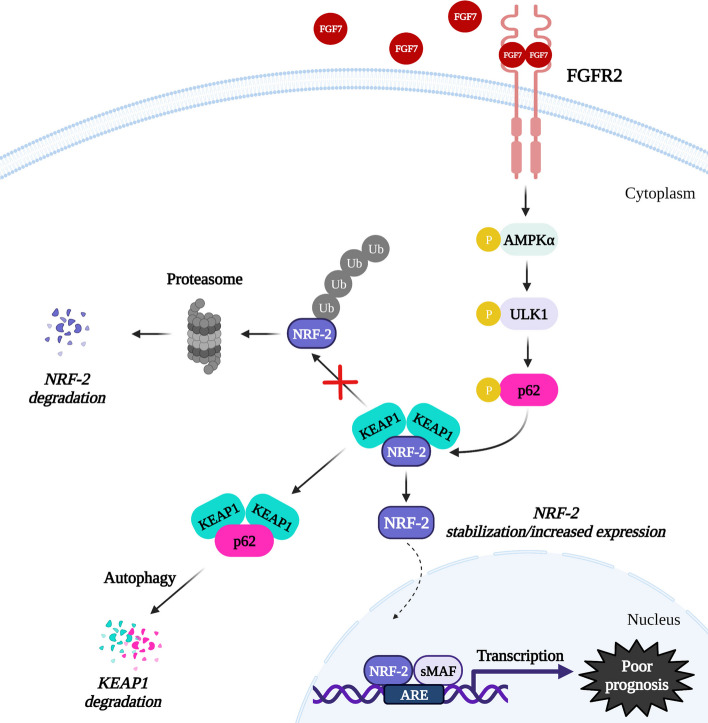

**Supplementary Information:**

The online version contains supplementary material available at 10.1186/s11658-024-00586-6.

## Background

Breast cancer (BCa) is the most commonly diagnosed cancer in women worldwide. Approximately 70–80% of BCa cases are classified as estrogen receptor‐positive (ER+) (luminal) subtype and targeting ER activity (i.e. tamoxifen or fulvestrant), the backbone of the first-line treatment, has significantly improved survival of patients with ER+ BCa. For instance, in early-stage ER+ BCa, 5-year adjuvant treatment with tamoxifen diminishes the 15-year mortality rate by about one-third [1]. However, despite the great efficacy of anti-ER drugs, resistance to the treatment poses a serious clinical problem. It has been documented that the resistance to anti-ER compounds develops in around 25% of early-stage and nearly in all metastatic BCa [2]. Therefore, there is a pressing need to identify the mechanisms contributing to development of resistance to the available treatment that, by providing the basis for the development of new therapeutic strategies, will improve BCa patients` survival. We and others have demonstrated that signalling mediated by FGFR is involved in the progression and resistance of BCa to anti-ER drugs [3–6] and hence, is a promising candidate for a ‘druggable’ target in ER+ BCa. However, our recent clinical analyses revealed that prognostic value of FGFR2 in BCa depends on the ‘hormonal context’. High expression of FGFR2 was found to be associated with longer disease-free and overall survival in ER+/PR+ BCa whereas this correlations are diminished in ER+/PR− cases [7]. We have also showed that the significance of FGFR2 in ER+/PR+ BCa patients depends on their menopausal status. The good prognostic effect of high expression of FGFR2 was lost in premenopausal BCa patients [8].

There is growing evidence identifying autophagy as one of the mechanisms promoting drug resistance in cancer, including ER+ BCa [9–15]. Macroautophagy (herein, referred to as autophagy) is a highly conserved, physiological process of degradation of cellular components within the lysosomes. Autophagy is triggered mainly by AMPKα-dependent ULK1 activation and inhibition of mTOR. The process involves formation of a unique double-membrane structure called autophagosome, its fusion with lysosome that leads to autolysosome formation and degradation of proteins and/or cellular components. The protein Sequestosome 1 (SQSTM1), also named p62, is an autophagy receptor and a selective substrate, as it delivers the cargo to autophagosomes and is finally degraded by autophagy. Furthermore, p62 is closely connected with the Keap1 (Kelch-like ECH-associated protein 1)/Nrf-2 (Nuclear factor E2-related factor 2) complex, one of the master regulators of antioxidant response [16, 17]. Under basal conditions, Keap1 mediates proteasomal degradation of Nrf-2 transcription factor. Upon oxidative stress, Keap1/Nrf-2 complex dissociates which is followed by nuclear translocation of Nrf-2 and activation of transcription of various cytoprotective genes e.g. *HMOX1*, *NQO1* and *SOD1*. Deregulation of p62/Keap1/Nrf-2 axis has been reported in various cancers [18–23]. Recent evidence suggests that Nrf-2 is involved in BCa resistance to anti-ER drugs [24–27]. Overexpression of Nrf-2 has been correlated with higher tumour aggressiveness and poorer survival of BCa patients [28–32].

Herein, we have analysed a potential contribution of the autophagy process and/or the p62/Keap1/Nrf-2 axis to the negative effect of FGFR2 on BCa cell response to anti-ER treatment. We have found that FGFR2 signalling regulated the expression level of autophagy markers (p62 and LC3B) and promoted autophagy flux. FGFR2 activity induced dissociation of Keap1/Nrf-2 complex and activation of Nrf-2. Both, FGFR2-dependent autophagy and Nrf-2 activation were found to counteract the effect of anti-ER drugs on BCa cell growth. Moreover, in silico analysis showed that high expression of *NFE2L2* (gene encoding Nrf-2) among patients with high *FGFR2* expression had a tendency to associate with shorter relapse-free survival (RFS), which suggests that Nrf-2 may determine the clinical significance of FGFR2 in BCa patients.

## Materials and methods

### Cell culture, antibodies and treatments

In the study, we used three ER-positive breast cancer cell lines, MCF7, T47D and CAMA-1, all expressing wild-type FGFR2, Keap1 and Nrf-2 [33, 34]. MCF7 and T47D cell lines were obtained from ATCC and DSMZ, respectively, and cultured in DMEM (Corning, NY, USA). CAMA-1 was purchased from ATCC and grown in MEM (Corning). All culture media were supplemented with 10% FBS (Biowest, Nuaillé, France), 100 U/ml penicillin and 100 μg/ml streptomycin (HyClone, Logan, UT, USA). Cells were maintained at 37 °C in an humidified atmosphere containing 5% CO_2,_ passaged for a maximum of 2–3 months, and routinely tested for mycoplasma contamination. The antibody against β-actin (A5316) was purchased from Sigma-Aldrich. The following antibodies were obtained from Cell Signaling Technology: anti-AMPKα (#5832), anti-phospho-AMPKα Thr172 (#2535), anti-FGFR1 (#9740), anti-FGFR2 (#23328), anti-FGFR3 (#4574), anti-FGFR4 (#8562), anti-Lamin B1 (#13435), anti-LC3B (#2775), anti-mTOR (#2983), anti-phospho-mTOR Ser2448 (#5536), anti-SQSTM1/p62 (#88588), anti-phospho-SQSTM1/p62 Ser349 (#16177), anti-phospho-SQSTM1/p62 Ser403 (#39786), anti-Raptor (#2280), anti-phospho-Raptor Ser792 (#2083), anti-ULK1 (#8359), anti-phospho-ULK1 Ser555 (#5869), anti-Vinculin (#13901). Anti-Keap1 (sc-365626) and anti-Nrf-2 (ab62352) antibodies were purchased from Santa Cruz Biotechnology and Abcam, respectively. The secondary anti-mouse (AlexaFluor® 790) and anti-rabbit (AlexaFluor® 680) antibodies were obtained from Jackson ImmunoResearch. For all treatments, FGF7 (50 ng/ml, PeproTech) was applied with heparin sodium salt (50 ng/ml; Sigma‐Aldrich). 4‐hydroxytamoxifen (4-OHT, an active metabolite of tamoxifen), fulvestrant (Fulv), MG132 and SBI-0206965 were purchased from Selleck Chemicals. Chloroquine diphosphate salt (CQ), K67 and *tert*-butylhydroquinone (tBHQ) were supplied from Sigma-Aldrich.

### Lentiviral transduction

For stable knock-down of FGFR2, MCF7 and T47D cells were transduced by lentiviral transfer of shRNA from Horizon discovery (RHS3979‐201732642, Dharmacon) and were called MCF7 FGFR2(−) and T47D FGFR2(−), respectively. MCF7 and T47D cells with Nrf-2 overexpression (MCF7 Nrf-2↑ and T47D Nrf-2↑) were generated by lentiviral transduction with pHAGE-NFE2L2 plasmid (#116765, Addgene). TRC Lentiviral shRNA plasmids against human SQSTM1/p62 (RHS3979-201739509, RHS3979-201739510, Horizon discovery, Dharmacon) were used to generate stable knock-down of p62 in MCF7 (MCF7 p62(−)^1^ and MCF7 p62(−)^2^) and T47D cells (T47D p62(−)^1^ and T47D p62(−)^2^). Cell lines with knock-down of individual protein were maintained in a medium supplemented with 0.2 μg/ml puromycin (Sigma‐Aldrich). Silencing of FGFR2 and p62 was confirmed by western blot analysis before each set of experiments, whereas cell line variants with Nrf-2 overexpression, which co-expressed GFP, were monitored by fluorescence microscopy. Corresponding empty vectors served as the negative control in all experiments.

### Cell proliferation assay

For cell proliferation studies, 5 × 10^3^ MCF7 or T47D cells were seeded in 96-well plates in triplicates and treated with DMSO or increasing concentrations of fulvestrant on the following day. After 72 h, the 3‐(4,5‐dimethylthiazol‐2‐yl)‐2,5‐diphenyltetrazolium bromide (MTT, Sigma‐Aldrich) solution (0.5 mg/ml) was added into each well and incubated for 2 h at 37 °C. Next, the medium was discarded and formazan crystals were dissolved in DMSO. The absorbance was measured at 590 nm.

### Three-dimensional cultures in Matrigel^®^

Three-dimensional cultures were performed in Matrigel® (growth factor reduced basement membrane matrix, Corning). T47D and CAMA-1 cells were trypsinized, counted and seeded (1.5 × 10^3^ and 3 × 10^3^ cells/well, respectively) to form Matrigel® drops with a volume of 40 µl (1:1 dilution of Matrigel® with culture medium) into 12-well tissue culture plates. After Matrigel® polymerization (20–30 min at 37 °C), a media containing appropriate inhibitors and/or growth factor were added to the wells. Cells were cultured for 14 days. Media were changed every 3 days. MCF7 cells, which do not form regular spheroids in Matrigel® drops, were cultured on Matrigel® “pillow”. For this purpose, 8-well chamber slides were covered with a layer of concentrated Matrigel® stock (100 µl) and allowed to polymerize for 20–30 min at 37 °C. Then, 5 × 10^3^ MCF7 cells/well were seeded, followed by 20–30 min incubation at 37 °C and media with appropriate inhibitors/growth factor and diluted Matrigel® (4% final concentration) were added to each well. Cells were cultured for 10–12 days. Media with 4% Matrigel® were replaced every 3 days. Cell growth was quantified by measuring the volume of 70–100 colonies/well using ZEISS PrimoVert microscope (Oberkochen, Germany) and ImageJ software.

### Western blot analysis

Cells were collected at 70–80% confluency and subjected to lysis with Laemmli buffer (2 × concentrated) containing 2 mM PMSF, 10 μg/ml aprotinin, 10 μg/ml leupeptin, 5 mM EGTA, 1 mM EDTA, 2 mM Na_4_P_2_O_7_, 5 mM NaF, and 5 mM Na_3_VO_4_. The subcellular fractions (cytoplasmic and nuclear) were isolated using REAP method [35]. An equal amount of extracted protein lysates (~ 20 μg) was separated using SDS/PAGE and transferred onto a nitrocellulose membrane. After blocking in 5% skimmed milk in TBS-T, the membranes were probed overnight with primary antibodies at 4 °C. Finally, following incubation with secondary antibodies conjugated with AlexaFluor® 790 or AlexaFluor® 680 (Jackson ImmunoResearch), the detected protein bands were visualized and quantified by Odyssey® CLx imaging system (LI-COR® Biosciences).

### Measurement of autophagy flux

Premo™ Autophagy Tandem Sensor RFP-GFP-LC3B Kit (Thermo Fisher Scientific) was used to monitor autophagy flux, according to the manufacturer’s instructions. MCF7 cells (3 × 10^4^ cells/well) were seeded on 8-well chamber slides, cultured overnight and then transfected with 9 µl of RFP-GFP-LC3B vector for 24 h. Cells were then exposed to FGF7 for 24 h and fixed with 4% paraformaldehyde. Cells were imaged with an OLYMPUS IX83 fluorescent microscope. The average number of autophagosomes—yellow dots (GFP+ /RFP+), autolysosomes—red dots (GFP−/RFP+) and calculation of the RFP/GFP ratio was used as an indicator of autophagic flux.

### Nrf-2 activity

Activity of Nrf-2 was determined in nuclear extracts of cells incubated for 6 and 24 h with FGF7 using Nrf2 Transcription Factor Assay Kit (Colorimetric, Abcam, ab207223), according to manufacturer’s protocols. The absorbance was measured at 450 nm.

### Proximity ligation assay (PLA)

For detection of Nrf-2 and Keap1 interactions, proximity ligation was performed with the Duolink™ In Situ PLA® Technology (Sigma‐Aldrich). Briefly, MCF7 and T47D cells were seeded in 8-well chamber slide, starved for 24 h and treated with FGF7 for 1 h. After fixation in 4% paraformaldehyde at room temperature (RT) and permeabilization with 0.1% Triton X‐100 at 4 °C, cells were blocked in 3% bovine serum albumin (BSA)/3% fetal bovine serum (FBS) solution in PBS for 1 h at RT and incubated overnight with primary antibodies at 4 °C. Washing, incubation with secondary antibodies and detection were done according to the manufacturer’s instruction. Cells’ nuclei were counterstained using Duolink® In Situ Mounting Medium with DAPI (Sigma‐Aldrich). Representative images were taken using OLYMPUS IX83 fluorescent microscope and Keap1/Nrf-2 complexes were quantified as the number of dots detected per cell by ImageJ software.

### Quantitative real-time PCR

The total RNA was extracted using PureLink™ RNA Mini Kit (Invitrogen), according to the manufacturer’s instructions. LunaScript® RT SuperMix Kit (New England Biolabs) was applied to cDNA synthesis. Quantitative PCR was performed using Luna Universal Probe qPCR Master Mix (New England Biolabs) and the following TaqMan probes (Life Technologies) were used: *HMOX1* (Hs01110250_m1), *NFE2L2* (Hs00975960_m1), *NQO1* (Hs00168547_m1), *SOD1* (Hs00533490_m1), *SQSTM1* (Hs00177654_m1). *ACTB* (Hs99999903_m1) and *GAPDH* (Hs02786624_g1) were applied as reference genes. Reactions were prepared in duplicates. Each plate contained a set of non‐template controls and controls for gDNA contamination. Gene expression was calculated according to the modified ΔΔC approach [36].

### Flow cytometry

Intracellular levels of reactive oxygen species (ROS) were measured by flow cytometry. MCF7 and T47D cells (2 × 10^5^) were placed in a 6 cm tissue culture dish. Cells were treated with FGF7 (50 ng/ml) for 30 and 60 min, *N*-acetyl-l-Cysteine (NAC; 5 mM) for 60 min or H_2_O_2_ (1 mM) for 30 min, followed by 15 min of staining (at 37 °C) with H_2_-DCFDA (10 μM). After that cells were washed with PBS, trypsinized, collected and resuspended in culture medium. Cells were subjected to cytometric analysis (CytoFLEX, Beckman Coulter) at λex = 488 nm, λem = 525/40 nm. Non-treated cells were used as a control, whereas NAC and H_2_O_2_ as negative and positive controls, respectively. Flow cytometry data were plotted and quantified, through the use of median fluorescence intensity. Three biological and three technical repeats were conducted. Statistical comparisons were made using one-way ANOVA and Dunnett's multiple comparisons tests.

### In silico analysis

In silico gene expression analysis was performed by utilizing data of the METABRIC cohort, publicly available from cBioPortal™ for Cancer Genomics (Breast Cancer, METABRIC dataset) [37, 38], as previously described [8]. mRNA levels of *FGFR2* and *NFE2L2* were determined using the expression microarray method and available clinicopathological characteristic was obtained. The following inclusion criteria were used: treatment‐naïve ER+ /PR+ invasive breast carcinomas of no special type (*n* = 825), reported menopausal status and follow‐up data (20 years). Tumours were dichotomized into groups with low/high levels of expression of the tested genes according to 1st tercile (*FGFR2*; cutoff previously established on clinical material at both RNA and protein levels [7, 8]) or median (*NFE2L2;* a distribution-based cutoff [0.172, with Youden's J statistic of 1.149] that is closest to the one identified with ROC analysis of RFS [0.180, with Youden's J statistic of 1.152]).

### Statistics

Data from three independent in vitro experiments are presented and expressed as the mean ± SD. An unpaired, 2-tailed Student’s *t*-test was used for two-group comparisons. *P*-values < 0.05 were considered as statistically significant. For in silico analyses, continuous data were presented as medians with interquartile ranges (IQR), whereas nominal data as numbers, followed by percentages in brackets. In case of non‐normal distribution according to the Shapiro–Wilk test, continuous variables were compared by the Mann–Whitney U‐test for two groups or the Kruskal–Wallis test (AKW; with Conover‐Inman post‐hoc test) for multiple groups. For normal distribution, Student's t‐test or one-/two‐way ANOVA (with Tukey’s post‐hoc test) was used. Differences in distribution of categorical variables were evaluated using Pearson's chi‐squared test or Fisher’s exact test, where applicable. The Kendall, Pearson’s and Spearman's correlation coefficients were calculated for correlations. Benjamini–Hochberg (BH) correction in case of multiple comparisons was applied. Receiver-operator-characteristic (ROC) analysis was used to dichotomize *NFE2L2* gene expression (AUC 0.577). Relapse‐free survival (RFS, the time from surgery to the time of recurrence or death) was presented using Kaplan–Meier curves and evaluated using Cox proportional hazard regression models. Data from in vitro experiments were analysed using GraphPad Prism (v.8.0.1, GraphPad Software) and in silico data were analysed using R (v.4.3.1).

## Results

### FGF7/FGFR2 signalling induces autophagy

**Fig. 1:**
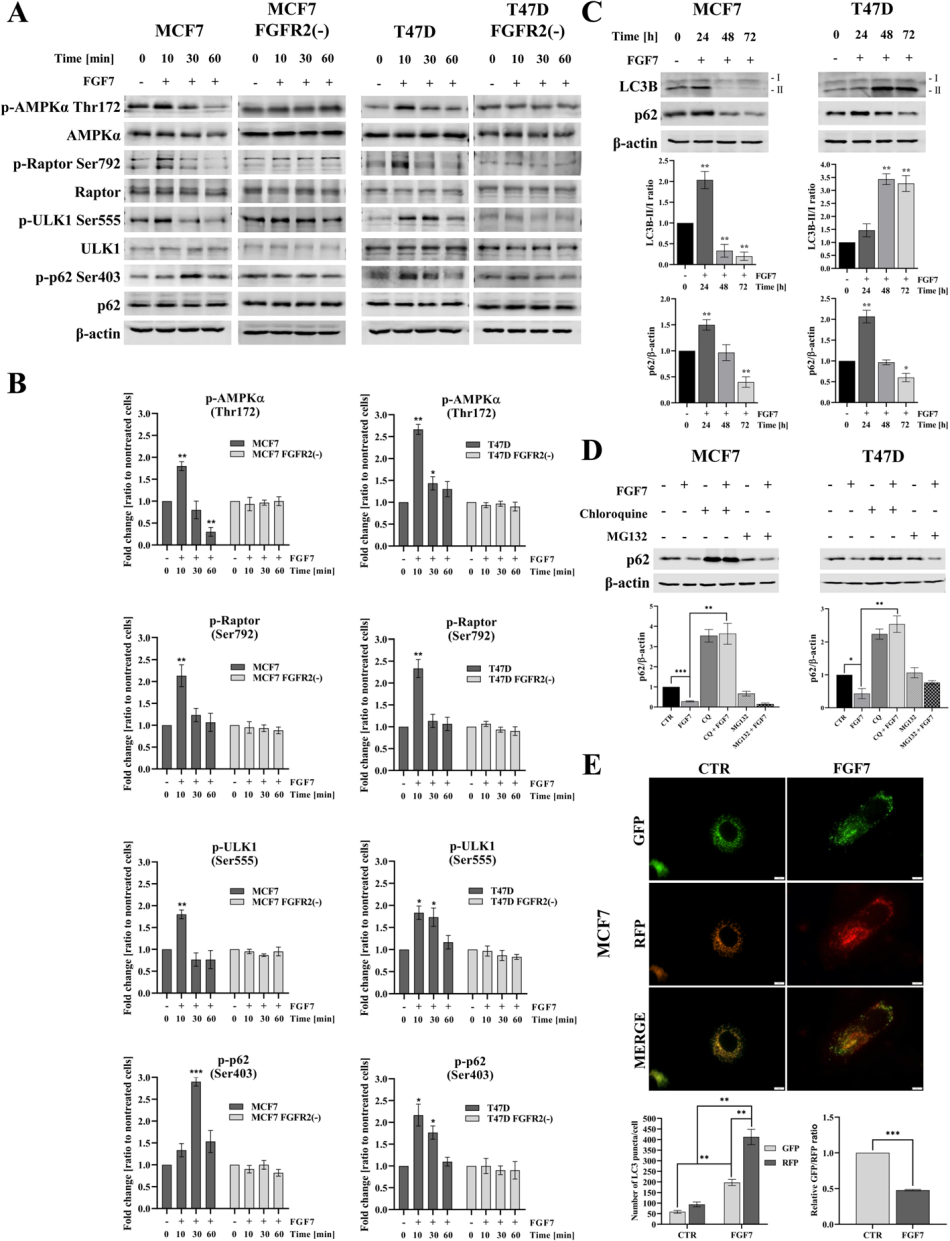
FGF7/FGFR2 signalling induces autophagy. **A**, **B** MCF7, MCF7 FGFR2(−),T47D and T47D FGFR2(−) cells were incubated with FGF7 (50 ng/ml) for 10, 30 and 60 min. AMPKα, Raptor, ULK1 and p62 phosphorylation was detected by western blotting and quantified by densitometry. Quantitative data are presented as the mean ± SD (*n* = 3), *P < 0.05, **P < 0.01 and ***P < 0.001. Statistical comparisons were made using 2-tailed Student’s *t*-test. **C** MCF7 and T47D cells were incubated with FGF7 (50 ng/ml) for 24–72 h. The quantification represents the relative p62/β-actin and LC3B-II/I expression levels, mean ± SD (*n* = 3), *P < 0.05, **P < 0.01. Statistical comparisons were made using 2-tailed Student’s *t*-test. **D** MCF7 and T47D cells were incubated for 72 h with the presence of FGF7 (50 ng/ml), ± CQ, (1 μM) ± MG132 (2 μM). p62 expression was analysed by western blotting and densitometry. Quantitative data are presented as the mean ± SD (*n* = 3), *P < 0.05, **P < 0.01 and ***P < 0.001. Statistical comparisons were made using 2-tailed Student’s *t*-test. **E** Quantification of LC3 positive puncta per cell (autophagosomes—yellow dots and autolysosomes—red dots) and GFP/RFP ratio in MCF7 cells transduced with RFP-GFP-LC3B vector and treated with FGF7 (50 ng/ml) for 24 h. Representative images were taken (scale bar 10 μm). Quantitative data are presented as the mean ± SD (*n* = 3), **P < 0.01 and ***P < 0.001. Statistical comparisons were made using 2-tailed Student’s *t*-test

Accumulating evidence demonstrated that autophagy promotes resistance to anti-ER drugs and BCa progression [9, 11, 15, 39–41]. On the other hand, our previous studies suggested that FGFR2 signalling counteracts the effect of tamoxifen in ER+ BCa [3, 6, 8]. To determine whether FGFR2 regulates autophagy, we analysed activation of the key regulators of the process in ER+ MCF7 and T47D cells treated with FGF7 (time course 0–60 min), a specific ligand of FGFR2 [42, 43]. We found that in both cell lines, FGF7 induced an increase in phosphorylation of AMPKα and ULK1 (Fig. 1A, B) as well as Raptor. AMPK-dependent phosphorylation of Raptor, which is a part of the mTOR complex, was previously shown to suppress mTOR activity [44]. Additionally, FGFR2 signalling enhanced phosphorylation of p62 at serine 403 previously suggested to promote the autophagic degradation of polyubiquitinated proteins [45]. To confirm the specificity of the observed effects for FGFR2 signalling, MCF7 and T47D cells with stable FGFR2 knock-down were used (called MCF7 FGFR2(−) and T47D FGFR2(−)) (Fig. S1A). Silencing of FGFR2 did not affect expression of other members of FGFR family. It was found that downregulation of FGFR2 abolished FGF7-triggered activation of the pathways involved in regulation of autophagy (AMPKα/ULK1) (Fig. 1A, B). To confirm the effect of FGFR2 on the induction of autophagy in cells treated with FGF7 (time course 0–72 h), we analysed the expression level of LC3B and p62, the commonly used markers of autophagy [46]. The results showed that although the kinetic of changes was different between MCF7 and T47D cells, FGF7 induced an increase in the LC3B-II/-I ratio (Fig. 1C) and a modest increase in p62 level upon 24 h (which may be caused by autophagosome formation) in both cell lines [46]. The latter was followed by consistent decrease in p62 level after 72 h incubation with FGF7, indicating autophagic degradation of p62 and completion of the process. Importantly, FGF7-triggered decrease in expression level of p62 was abrogated by knock-down of FGFR2 (Fig. S1B), suggesting that FGF7/FGFR2 signalling induces autophagy. Silencing of FGFR2 also resulted in a reduced level of p62. Although the effect was cell-specific (observed only in MCF7 cells) this supports an involvement of FGFR2 in regulation of autophagy and may originate from autocrine-dependent activation of the receptor. The role of FGFR2 in induction of autophagy has been verified by the application of chloroquine (CQ, late-phase autophagy inhibitor) which abolished FGF7-dependent decrease in p62 expression level (Fig. 1D). In contrast, incubation of the cells with proteasome inhibitor (MG132) did not rescue the expression level of p62. These may indicate that, in the presence of FGF7, p62 was degraded in an autophagy-dependent manner but not the ubiquitin–proteasome pathway. We have also analysed the autophagic activity (referred to as autophagy flux) by monitoring the maturation of the autophagosome into autolysosome. MCF7 cells were transduced with a RFP-GFP-LC3B tandem construct, known to distinguish autophagosomes (positive for both GFP and RFP and observed as yellow vehicles) from autolysosomes (positive only for RFP, as the GFP signal is quenched in the acidic environment of the lysosomes). As expected, stimulation with FGF7 increased the number of both autophagosomes and autolysosomes, with a significant predominance of the latter (Fig. 1E, left graph). The decrease in the GFP/RFP ratio confirmed induction of the autophagic flux by FGF7 (Fig. 1E, right graph). Collectively, these results demonstrated that FGF7/FGFR2 signalling induced autophagy.

### FGF7-induced autophagy abrogates the effect of anti-ER drugs

**Fig. 2:**
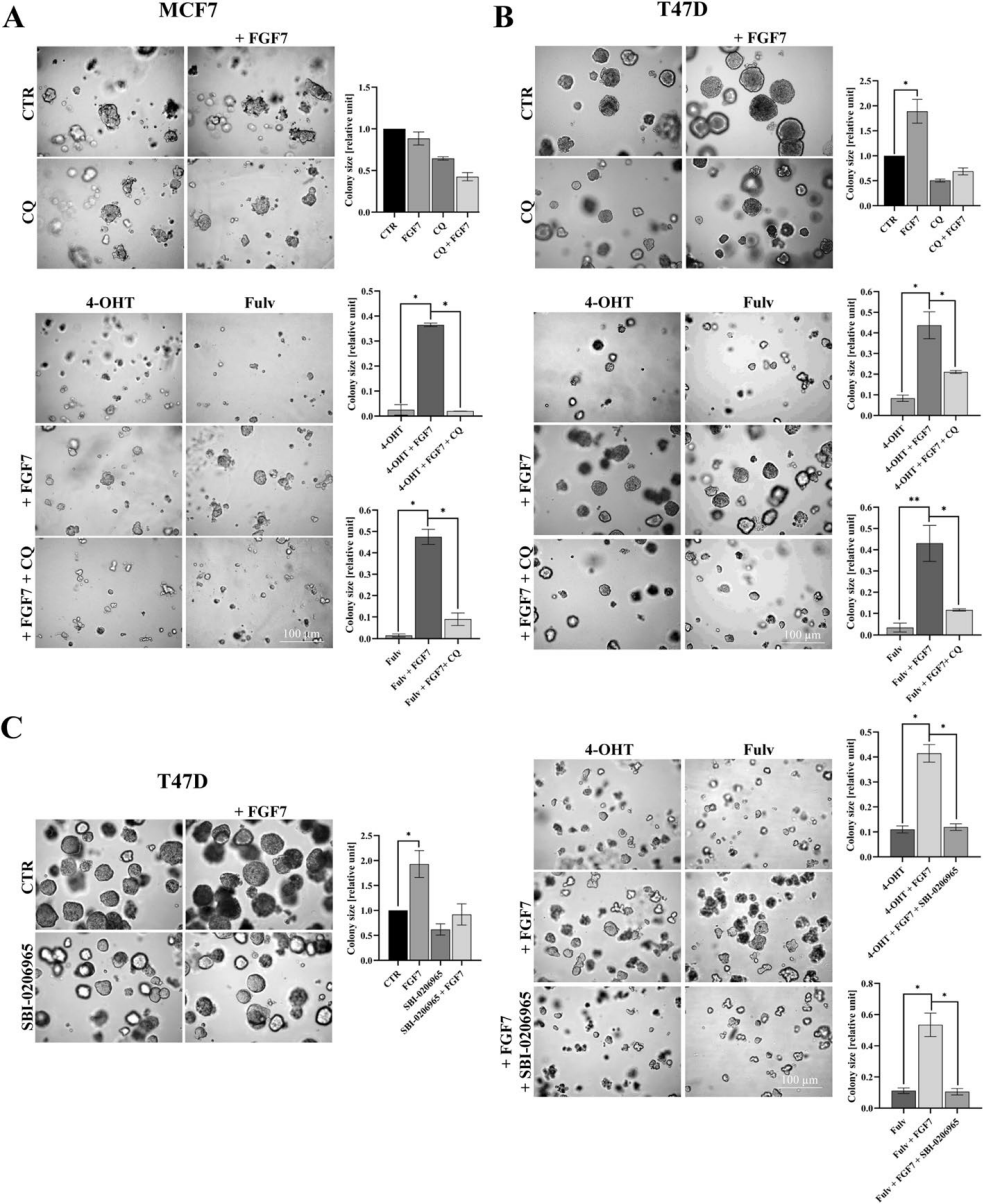
FGF7-induced autophagy abrogates the effect of anti-ER drugs. **A** MCF7 and **B** T47D cells were cultured in 3D Matrigel® for 10–12 days and 14 days, respectively, with FGF7 (50 ng/ml), ± 4-OHT (1 μM), ± Fulv (100 nM), ± CQ (1 μM). **C** T47D cells were cultured in 3D Matrigel® for 14 days with FGF7 (50 ng/ml) ± 4-OHT (1 μM), ± Fulv (100 nM), ± SBI-0206965 (750 nM). Representative images were taken (scale bar 100 μm), colonies were measured and analysed with ImageJ software. All quantitative data are presented as a relative ratio to CTR/non‐treated wild‐type cells, mean ± SD (*n* = 3), *P < 0.05 and **P < 0.01. All statistical comparisons were made using 2-tailed Student’s *t*-test

We have previously shown that FGFR2 signalling promotes ER+ BCa cell growth and resistance to tamoxifen [3, 8, 47]. Herein, we evaluated whether autophagy is involved in the FGFR2-driven counteraction of the effect of anti-ER drugs—tamoxifen (4-OHT) and fulvestrant (Fulv) on BCa cell growth. The results showed that inhibition of autophagy with CQ significantly impaired FGFR2-promoted MCF7 and T47D cells 3D growth in the presence of tamoxifen or fulvestrant (Fig. 2A, B). This has been confirmed with CAMA-1 cells, the third cell line used in our ER+ BCa model (Fig. S2). Moreover, the protective effect of FGF7 on anti-ER drugs was not observed in T47D FGFR2(−) cells (Fig. S3A). Next, assuming that the AMPKα/ULK1 cascade (Fig. 1A, B) might be crucial for FGFR2-promoted autophagy, we assessed an impact of inhibition of this pathway and hence, a blockage of autophagy, on restoration BCa cell sensitivity to anti-ER drugs. As expected, in the presence of FGF7, an addition of the AMPKα/ULK1 inhibitor (SBI-0206965) re-sensitized T47D cells to anti-ER drugs (Fig. 2C), which was abolished by knock-down of FGFR2 (Fig. S3B). Taken together, these results revealed that FGF7/FGFR2-induced autophagy affects BCa cell response to anti-ER drugs and indicated that this activation occurred in an AMPKα/ULK1-dependent manner.

### FGF7/FGFR2 signalling promotes dissociation of Keap1/Nrf-2 complex and activation of Nrf-2

**Fig. 3:**
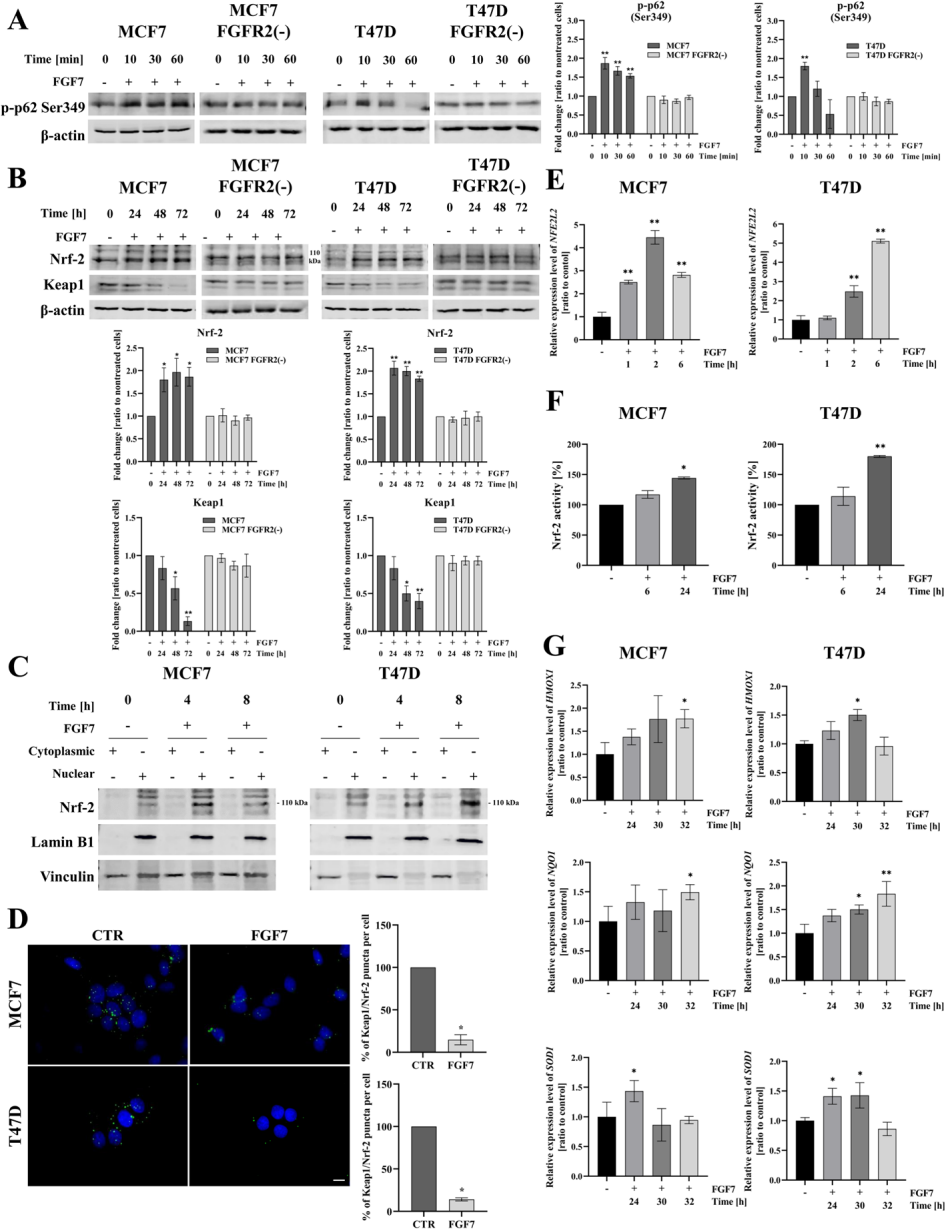
FGF7/FGFR2 signalling promotes dissociation of Keap1/Nrf-2 complex, Nrf-2 expression and activity. **A** MCF7, MCF7 FGFR2(−), T47D and T47D FGFR2(−) cells were incubated with FGF7 (50 ng/ml) for 10–60 min. p62 serine 349 phosphorylation was evaluated by western blotting and quantified by densitometry. Quantitative data are presented as the mean ± SD (*n* = 3), **P < 0.01. Statistical comparisons were made using 2-tailed Student’s *t*-test. **B** MCF7, MCF7 FGFR2(−), T47D and T47D FGFR2(−) cells were incubated with FGF7 (50 ng/ml) for 24, 48 and 72 h. The quantification represents the relative Nrf-2 and Keap1 expression levels normalized to β-actin, mean ± SD (*n* = 3), *P < 0.05, **P < 0.01. Statistical comparisons were made using 2-tailed Student’s *t*-test. **C** The expression level of Nrf-2 was determined by western blot analysis in cytoplasmic and nuclear extracts of MCF7 and T47D cells treated with FGF7 (50 ng/ml) for 4 and 8 h. Vinculin and Lamin B1 were used as loading controls for the cytoplasmic or nuclear fractions, respectively. **D** Detection of Nrf-2 and Keap1 interactions in MCF7 and T47D cells treated with FGF7 (50 ng/ml) for 1 h by proximity ligation assay. Representative fluorescent microscopy images were taken (scale bar 10 μm). Quantitative analysis of PLA results was done using ImageJ software and presented as % of Keap1/Nrf-2 puncta per cell, mean ± SD (*n* = 3), *P < 0.05. Statistical comparisons were made using 2-tailed Student’s *t*-test. **E** MCF7 and T47D cells were incubated with FGF7 (50 ng/ml) for 1, 2 and 6 h. The mRNA expression level of *NFE2L2* (gene encoding Nrf-2) was analyzed by qPCR. Data are shown as a ratio to control, mean ± SD (*n* = 3), **P < 0.01. Statistical comparisons were made using 2-tailed Student’s *t*-test. **F** MCF7 and T47D cells were treated with FGF7 (50 ng/ml) for 6 and 24 h, and nuclear extracts were analyzed for Nrf-2 binding capacity to antioxidant response element (ARE) by Nrf2 Transcription Factor Assay Kit. Data are shown as a ratio to control, mean ± SD (*n* = 3), *P < 0.05, **P < 0.01. Statistical comparisons were made using 2-tailed Student’s *t*-test. **G** MCF7 and T47D cells were incubated with FGF7 (50 ng/ml) for 24, 30 and 36 h, and mRNA levels of Nrf-2-dependent genes (*HMOX1, NQO1, SOD1*) were determined by qPCR. Data are shown as a ratio to control. All quantitative data are presented as the mean ± SD (*n* = 3), *P < 0.05, **P < 0.01. Statistical comparisons were made using 2-tailed Student’s *t*-test

It has been previously reported that phosphorylation of p62 at serine 349 enhances its binding with Keap1 which leads to dissociation of the Keap1/Nrf-2 complex and activation of Nrf-2 [16, 17]. The transcription factor Nrf-2 serves as a master regulator of cell response to oxidative stress and its activity is tightly controlled by the negative regulator Keap1. Since FGF7/FGFR2 signalling was found to induce phosphorylation of p62 at serine 349 (Fig. 3A), reported to induce Nrf-2 activation, we went on to investigate whether FGFR2 participates in the regulation of Keap1/Nrf-2 complex. We have found that stimulation with FGF7 upregulated and downregulated the level of expression of Nrf-2 and Keap1, respectively, only in FGFR2-positive but not FGFR2-negative MCF7 and T47D cells (Fig. 3B). There is a number of studies which discussed an issue of Nrf-2 detection by western blotting. It has been established that Nrf-2 migrates at ~ 110 kDA and additional observed bands reflect post-translational modifications of Nrf-2, alternative splicing of Nrf-2 coding gene or partial cleavage of the protein [48–50]. To confirm the specificity of the anti-Nrf-2 antibody, MCF7 and T47D cells were treated with tBHQ (*tert*-butylhydroquinone)—Nrf-2 inductor, which was previously shown to induce Nrf-2 protein expression (51–53). Incubation with tBHQ resulted in increased intensity only of the band at approximately 110 kDa (Fig. S4). In the presence of FGF7, we showed an increase of Nrf-2 in the nuclear fraction of the cell lysates, highly suggestive of FGF7-triggered activation of Nrf-2 (Fig. 3C). No effect of FGF7 treatment on Nrf-2 expression in the nuclear fraction of MCF7 FGFR2(−) and T47D FGFR2(−) was observed (Fig. S5). To analyse the interaction of Keap1 with Nrf-2, we used the proximity ligation assay (PLA) which showed that treatment with FGF7 led to a significantly decreased number of Keap1/Nrf-2 complexes (Fig. 3D). To verify whether FGFR2-dependent dissociation of Keap1/Nrf-2 complexes is the result of reactive oxygen species (ROS) production, we measured intracellular ROS levels by H_2_-DCFDA (indicator for ROS in cells) staining. There were no remarkable changes found in the generation of ROS upon stimulation of MCF7 and T47D cells with FGF7 (Fig. S6), which suggests an alternative pathway inducing the dissociation of Keap1/Nrf-2 complex. Next, we demonstrated that FGF7-induced upregulation of the mRNA level of *NFE2L2* (Nrf-2 coding gene) (Fig. 3E) coincided with increased Nrf-2 activity (Fig. 3F) and enhanced expression of Nrf-2-regulated genes i.e. *HMOX1* (Heme Oxygenase 1), *NQO1* (NAD(P)H Quinone Dehydrogenase 1) and *SOD1* (Superoxide Dismutase 1) (Fig. 3G). Taken together, these results suggest that FGF7/FGFR2 signalling triggers ROS-independent dissociation of Keap1/Nrf-2 complex, that is followed by Nrf-2 activation.

### p62/Keap1/Nrf-2 axis is involved in FGFR2-dependent negative cell response to anti-ER drugs

**Fig. 4:**
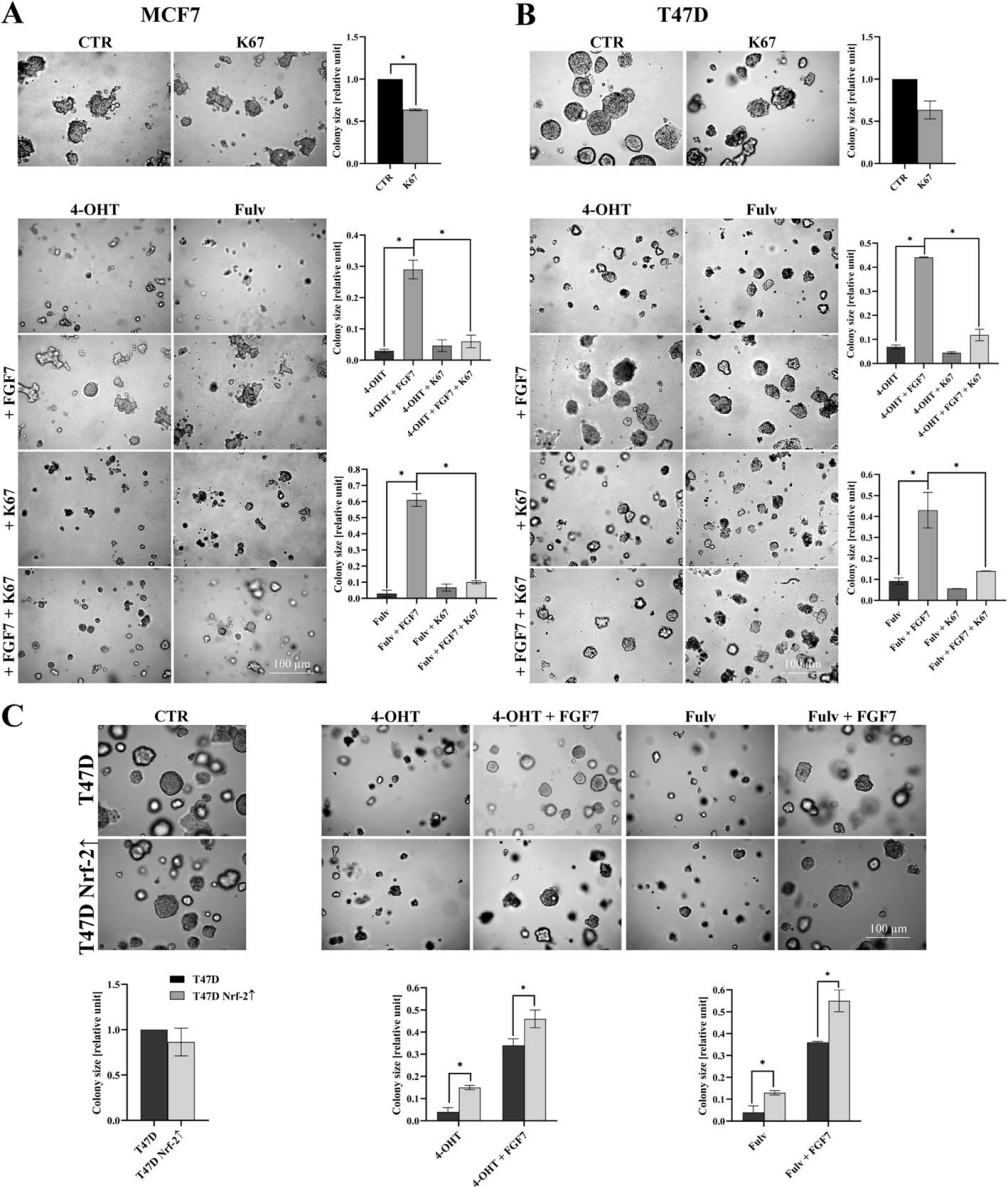
Nrf-2 is involved in FGFR2-dependent response to anti-ER drugs. **A** MCF7 and **B** T47D cells were cultured in 3D Matrigel® for 10–12 days and 14 days, respectively, with FGF7 (50 ng/ml), ± 4-OHT (1 μM), ± Fulv (100 nM), ± K67 (500 nM). Representative images were taken (scale bar 100 μm), colonies were measured and analysed with ImageJ software. Quantitative data are presented as a relative ratio to CTR/non‐treated wild‐type cells, mean ± SD (*n* = 3), *P < 0.05. Statistical comparisons were made using 2-tailed Student’s *t*-test. **C** T47D and T47D Nrf-2↑ cells were cultured in 3D Matrigel® for 14 days with FGF7 (50 ng/ml) ± 4-OHT (1 μM) ± Fulv (100 nM). Representative images were taken (scale bar 100 μm), colonies were measured and analysed with ImageJ software. All quantitative data are presented as a relative ratio to CTR/non‐treated wild‐type cells, mean ± SD (*n* = 3), *P < 0.05. Statistical comparisons were made using 2-tailed Student’s *t*-test

To examine whether FGFR2-mediated activation of Nrf-2 affects cell response to tamoxifen and fulvestrant, MCF7 and T47D cells were cultured in the presence of K67, an Nrf-2 inhibitor. K67 has been reported to inhibit an interaction between Keap1 and phosphorylated serine 349 of p62 leading to Keap1-mediated degradation of Nrf-2 [54]. Analysis of 3D growth demonstrated that degradation/inhibition of Nrf-2 abrogated the effects of FGF7 and restored MCF7 and T47D cells` sensitivity to anti-ER drugs (Fig. 4A, B). Similar results were obtained for the CAMA-1 cell line (Fig. S7). The specificity of the observed results for FGF7/FGFR2 signalling was confirmed in the T47D FGFR2(−) cell line (Fig. S8). To further explore the role of Nrf-2 in BCa cell response to anti-ER drugs, we have developed MCF7 and T47D cell line variants with stable overexpression of Nrf-2 (MCF7 Nrf-2↑ and T47D Nrf-2↑, respectively) (Fig. S9A, B). Interestingly, Nrf-2 overexpression resulted in an elevated p62 protein level, as well as an increased *SQSTM1* mRNA, a gene encoding p62 (Fig. S9C, D). These results are consistent with the published data, implying a positive feedback loop, where on one side p62 mediates activation of Nrf-2 on the other Nrf-2 positively regulates *SQSTM1* expression (55). As a result Nrf-2 can regulates its own expression. More importantly, we found that increased expression of Nrf-2 significantly reduced cell sensitivity to anti-ER drugs and enhanced FGF7-mediated protective effect towards tamoxifen and fulvestrant both in 3D (Fig. 4C) and classical 2D cell proliferation assay (Fig. S9E, F). These data collectively suggest that FGFR2-dependent activation of Nrf-2 counteracts the effect of anti-ER drugs on BCa cell growth.

**Fig. 5:**
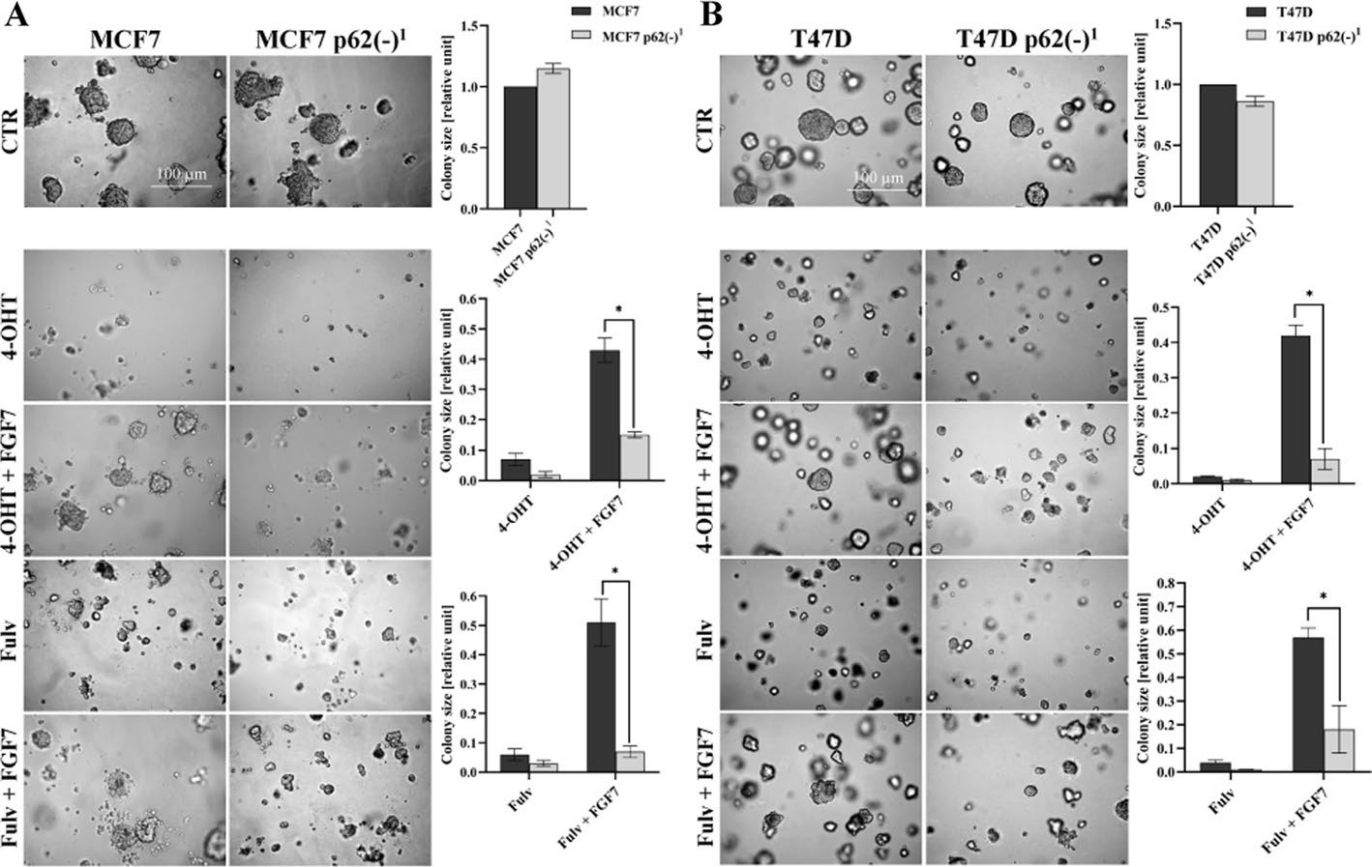
Knock-down of p62 re-sensitized FGF7-treated cells to anti-ER drugs. **A** MCF7, MCF7 p62(−)^1^ and **B** T47D, T47D p62(−)^1^ cells were cultured in 3D Matrigel® for 10–12 days and 14 days, respectively, with FGF7 (50 ng/ml), ± 4-OHT (1 μM), ± Fulv (100 nM). Representative images were taken (scale bar 100 μm), colonies were measured and analysed with ImageJ software. All quantitative data are presented as a relative ratio to CTR/non‐treated wild‐type cells, mean ± SD (*n* = 3), *P < 0.05. Statistical comparisons were made using 2-tailed Student’s *t*-test

To determine whether p62 is involved in Nrf-2-mediated BCa cell protection from anti-ER drugs, we used two specific shRNA constructs to develop p62 knock-down in MCF7 (Fig. S10A) and T47D cells (Fig. S10B). Analysis of 3D cell growth showed that loss of p62 abolished the protective effect of FGF7 for both tamoxifen and fulvestrant in MCF7 p62(−)^1^ and T47D p62(−)^1^ cells (Fig. 5A, B). Similar results were obtained for the second variant of p62 knock-down in MCF7 p62(−)^2^ and T47D p62(−)^2^ cell lines (data not shown). Engagement of p62 in the protective effect of FGF7 from fulvestrant was further confirmed in the proliferation assay (Fig. S10C, D). Overall, these results confirmed that FGFR2-dependent activation of p62/Keap1/Nrf-2 axis might be involved in BCa cell resistance to anti-ER treatment.

### Expression of *NFE2L2 *affects the prognostic value of *FGFR2* in ER+ BCa

**Fig. 6:**
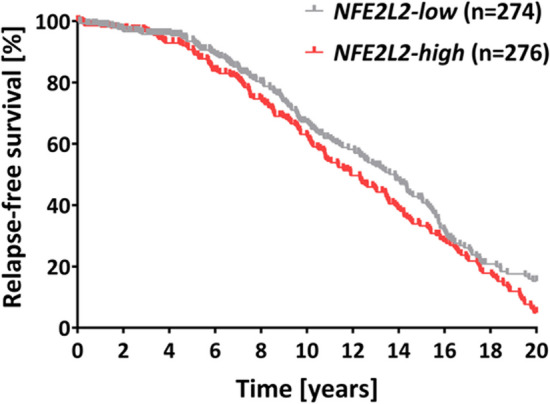
Expression of *NFE2L2* affects the prognostic value of FGFR2 in ER+ /PR+ BCa. Kaplan–Meier curve for relapse-free survival (RFS) in METABRIC cohort of ER+ BCa patients (*n* = 825). Samples were divided into *FGFR2*-low and *FGFR2*-high subgroups. *FGFR2*-high subgroup was further subdivided based on *NFE2L2* gene expression status by median to *NFE2L2*-low (*n* = 274) and *NFE2L2*-high (*n* = 276). *P*‐value was calculated using the Cox proportional hazard regression models (HR = 1.278; 95% CI 1.026–1.593; *P* = 0.029)

We have recently shown that the clinical significance of FGFR2 in BCa is context-dependent i.e. high expression of FGFR2 is a good prognostic factor in ER+/ PR+ whereas this association is lost in ER+/PR− BCa [7]. To determine if Nrf-2 correlates with FGFR2 and/or combination of the two alters the prognostic value of the latter, we performed in silico analysis in the group of 825 patients from METABRIC dataset (as described in our previous study) [8]. The clinicopathological features of BCa patients are summarized in the Supplementary Table 1. In the METABRIC group there was no significant correlation between *NFE2L2* and *FGFR2* gene expression, as demonstrated by three different correlation coefficients (Kendall’s: *R* = 0.05; *p*-value = 0.031; Pearson’s: *R* = 0.09; *p*-value = 0.014; Spearman’s: *R* = 0.08; *p*-value = 0.031). Out of 825 BCa patients from the METABRIC database, 550 were classified as *FGFR2*-high (Supplementary Table 2). The *FGFR2*-high group was further divided based on the *NFE2L2* expression status into *NFE2L2*-low = 274 and *NFE2L2*-high = 276 (Supplementary Table 3). To examine the clinical value of Nrf-2, we investigated the associations between the level of *NFE2L2* mRNA and clinicopathological features (i.e. tumor size, histological subtype, tumour grade, stage, HER2 status or age at diagnosis). Among clinicopathological features only age at diagnosis showed significant difference i.e. *NFE2L2*-high patients displayed lower age at diagnosis compared to *NFE2L2*-low patients (median 63 years vs 66 years, respectively) (Fig. S11). Importantly, expression of *NFE2L2* was found to affect an association of *FGFR2* with good prognosis i.e. *FGFR2*-high/*NFE2L2*-high patients tended to display shorter relapse-free survival (RFS) than *FGFR2*-high*/NFE2L2*-low patients (HR = 1.278; 95% CI 1.026–1.593; *p* = 0.029) (Fig. 6). Taken together, these results suggest that, in a subgroup of BCa patients, expression of *NFE2L2* may influence clinical significance of *FGFR2*.

## Discussion

Multiple studies, including our work, confirmed the involvement of FGF/FGFR signalling in BCa progression and resistance to anti-ER drugs [5]. Abnormalities in the FGFR signalling pathway, including genetic aberrations, occur in 40% of BCa patients resistant to anti-ER therapy, which puts forward FGFR as a potential target candidate for therapeutic strategies [4]. Interestingly patients' response to inhibition of FGFR is highly variable and does not depend solely on the status of the receptors i.e. amplification, overexpression or mutation. Identification of patients who can benefit from anti-FGFR treatment poses a serious clinical challenge. Zingg et al. found that E18-truncated variant of *FGFR2* (*FGFR2*^*ΔE18*^) acts as a potent single-driver alteration in cancers, whereas the tumourigenic abilities of full-length *FGFR2* variant depend on cooperating driver genes [56]. Patients with *FGFR2*^*ΔE18*^ displayed favourable responses to anti-FGFR therapy over those with other FGF/FGFR alterations. Therefore, co-mutational landscapes/other markers should be considered during the selection of patients for anti-FGFR therapies. This has been supported by our analyses indicating that the prognostic value of FGFR2 in BCa is context-dependent [7, 8].

Autophagy has been distinguished as one of the mechanisms interconnected with tumour microenvironment, responsible for promotion of cancer cell survival and resistance to applied therapy. Increasing evidence indicates plausible asset of autophagy inhibition in BCa cells with acquired resistance to anti-ER drugs [10, 12–14, 57]. So far, several independent groups described the role of FGFRs in the regulation of autophagy [58–61] and these appear to be context-dependent and tumour-specific. For instance, Nanni et al. showed that FGFR2 and its downstream substrate JNK1 are involved in the activation of autophagy and differentiation of keratinocytes. On the other hand, Yuan et al. demonstrated that FGFR1 signalling inhibits autophagy in FGFR1-amplified non-small cell lung cancer cells. To the best of our knowledge, the involvement of FGFRs in the regulation of autophagy in breast cancer cells and, in particular, in the relation to the response to anti-ER drugs, has not been yet investigated. Taking into consideration that FGFR2 signalling, as well as autophagy have been demonstrated to contribute to poor response to anti-ER therapy, here, we evaluated the potential link between the two mechanisms in ER+ BCa. Here, we showed for the first time that FGF7/FGFR2 signalling induced autophagy in AMPKα/ULK1-dependent manner and inhibition of autophagy, as well as AMPKα/ULK1 counteracted FGFR2-mediated protective effect to anti-ER treatment. In recent studies, AMPKα inhibition has been also shown to sensitize BCa cells to tamoxifen and endoxifen [11]. This may have important implication, as availability of autophagy inhibitors is limited (no significant efficacy of chloroquine or hydroxychloroquine as a single agent in BCa), an inhibition of upstream mediators of autophagy can be a promising therapeutic approach [62]. A few ongoing clinical trials investigate hydroxychloroquine in combination with mTOR kinase inhibitor, aromatase inhibitor and/or CDK4/6 inhibitors [63].

Autophagy has been linked with the master mediator of antioxidant response, namely, the Keap1/Nrf-2 complex. A p62, the autophagy receptor and a selective substrate, promotes Nrf-2 activation by Keap1/Nrf-2 complex dissociation [61, 64]. A finding that FGFR2-mediated p62 serine 349 phosphorylation (responsible for dissociation of Keap1/Nrf-2 complex and activation of Nrf-2) prompted us to investigate whether FGFR2 participates in the regulation of the abovementioned complex. We demonstrated that FGFR2 signalling triggers ROS-independent Keap1/Nrf-2 complex dissociation, upregulates both Nrf-2 level and expression of Nrf-2-dependent genes. Nrf-2 was previously shown to autoregulate its expression [65]. The observed FGFR2-mediated increase in the level of Nrf-2 protein and its gene (mRNA level of *NFE2L2*) suggest autoregulation of Nrf-2 expression which was reflected in enhanced Nrf-2 activity. Interestingly, p62 has been shown to form cytoplasmic protein aggregates, called p62 droplets/bodies, created by liquid–liquid phase separation [66–68]. In addition, p62 bodies contain p62-binding partners, such as ubiquitinated proteins, targeted for autophagic degradation. Ikeda et al. have demonstrated that ULK1 phosphorylates Ser349 of p62 located in p62 bodies, which promotes the recruitment of Keap1, followed by activation of Nrf-2 [69]. Although we did not investigate the formation of p62 bodies, observed FGFR2-dependent phosphorylation of p62 and ULK1 suggest that this could be a potential mechanism of Nrf-2 activation.

Furthermore, we demonstrated that Nrf-2 was involved in FGFR2-promoted poor response of BCa cell to anti-ER drugs. This is in agreement with other studies indicating an involvement of Nrf-2 in the development of resistance to anti-ER therapy. Recent data identify various mechanisms of Nrf-2-dependent anti-ER drugs resistance including higher antioxidant capacity Recent data indicate various mechanisms of Nrf-2-dependent anti-ER drugs resistance including higher antioxidant capacity [27], increased expression of antioxidant genes (*NQO1*, *HMOX1*, *SOD1*) or multi-drug resistance transporters (MDRTs) [24, 25]. Our findings demonstrating that FGFR2 activation leads to an increased expression of antioxidant genes, broaden the repertoire of molecular events contributing to the development of resistance to anti-ER therapy and may have important clinical implications. Moreover, overexpression of Nrf-2 resulted in elevated protein and mRNA level of p62, suggesting that p62 and Nrf-2 are sustained in a positive feedback loop, which is in line with published data [55, 64]. As expected, depletion of p62 abolished the protective effect of FGF7 for anti-ER drugs, which indicates that FGFR2-dependent activation of p62/Keap1/Nrf-2 axis might contribute to BCa cell resistance to anti-ER drugs. Further in vivo studies are required to confirm the observed involvement of FGFR2-mediated activation of p62/Keap1/Nrf-2 axis in developing resistance to anti-ER therapy.

The final stage of the study involved in silico evaluation of the potential prognostic value of the FGFR2/Nrf-2 interdependence in ER+ BCa samples. High expression of *NFE2L2* among *FGFR2*-high patients was found to display shorter RFS than *NFE2L2*-low patients. However, to fully uncover the significance of FGFR2→Nrf-2 axis for ER+ BCa patients the examination of the FGFR2/Nrf-2 correlation at the protein level (determined immunohistochemically), cellular location of Nrf-2 and its activity, e.g. evaluation of Nrf-2-dependent genes expression in relation to other clinicopathological factors, known to affect disease outcome, should be taken into consideration. The more in-depth clinical analyses are beyond the framework set by the aims of the presented study. Importantly, our results are consistent with our previous observations and reveal another level of heterogeneity of BCa that might have clinical implications as determination of *NFE2L2* expression can prove useful for identification of FGFR2-positive patients with a poor prognosis.

## Conclusions

**Fig. 7:**
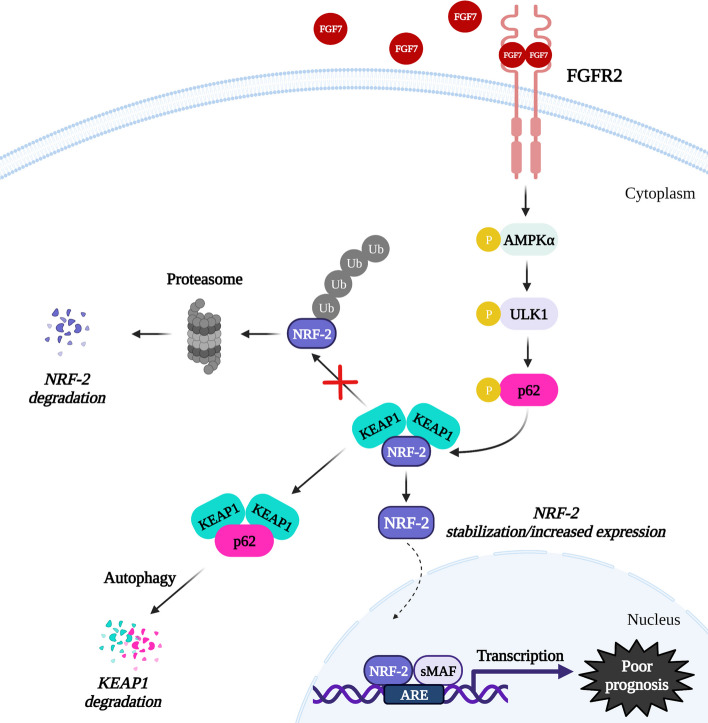
Predicted mechanism of FGFR2-dependent regulation of p62/Keap1/Nrf-2 axis in ER+ BCa. FGF7/FGFR2 signalling induces autophagy in AMPKα/ULK1-dependent manner. FGFR2-mediated p62 serine 349 phosphorylation leads to Keap1/Nrf-2 complex dissociation, followed by Nrf-2 stabilization/increased expression resulted in higher activity of Nrf-2, which might be involved in BCa cell resistance to anti-ER treatment and poor prognosis of patients’. Created with BioRender.com

This study uncovered the unknown role of FGFR2 signalling in activation of autophagy and regulation of the p62/Keap1/Nrf-2 interdependence (a proposed model of FGFR2 action is presented in Fig. 7) with a negative impact on the response of ER+ BCa cells to anti-ER therapies. The data from in silico analyses emphasize the fact that prognostic value of FGFR2 needs to be analysed in relation to another molecular factors important for BCa pathophysiology. It seems that expression of Nrf-2 could act as a marker indicating potential benefits of implementation of anti-FGFR therapy in patients with ER+ BCa, in particular, when applied in combination with anti-ER drugs.

### Supplementary Information

Supplementary Material 1. Supplementary Figure S1. FGFR2 mediates FGF7-dependent effect on the expression of p62. (A) Knock-down of FGFR2 and the expression levels of FGFR1, FGFR3 and FGFR4 were verified by western blotting in MCF7 and T47D cells. (B) Evaluation of p62 expression level by western blotting in MCF7, MCF7 FGFR2(−), T47D and T47D FGFR2(−) cells treated with FGF7 (50 ng/ml) for 24 h. All quantitative data are presented as the mean ± SD (*n* = 3), **P < 0.01. Statistical comparisons were made using 2-tailed Student’s *t*-test.

Supplementary Material 2. Supplementary Figure S3. Inhibition of autophagy restores sensitivity to anti-ER drugs in CAMA-1 cell line. CAMA-1 cells were grown in 3D Matrigel® for 14 day in the presence of FGF7 (50 ng/ml), ± 4-OHT (1 μM), ± Fulv (100 nM), ± CQ (1 μM). Representative images were taken (scale bar 100 μm), colonies were measured and analysed with ImageJ software. All quantitative data are presented as a relative ratio to CTR/non‐treated wild‐type cells, mean ± SD (*n* = 3), *P < 0.05 and **P < 0.01. Statistical comparisons were made using 2-tailed Student’s *t*-test.

Supplementary Material 3. Supplementary Figure S3. Knock-down of FGFR2 abolishes the effects of FGF7-induced autophagy. (A) T47D FGFR2(−) cells were cultured in 3D Matrigel^®^ for 14 days with FGF7 (50  ng/ml), ± 4-OHT (1  μM), ± Fulv (100 nM), ± CQ (1  μM). (C) T47D FGFR2(−) cells were cultured in 3D Matrigel^®^ for 14 days with FGF7 (50 ng/ml)  ± 4-OHT (1 μM), ± Fulv (100 nM), ± SBI-0206965 (750 nM). Representative images were taken (scale bar 100  μm), colonies were measured and analysed with ImageJ software. All quantitative data are presented as a relative ratio to CTR/non‐treated wild‐type cells, mean ± SD (*n* = 3), ns: not significant. All statistical comparisons were made using 2-tailed Student’s *t*-test.

Supplementary Material 4. Supplementary Figure S4. Induction of Nrf-2 expression by tBHQ. To establish the specificity of anti-Nrf-2 antibody, MCF7 and T47D cells were treated for 24 h with tBHQ (50 μM). Nrf-2 expression was analysed by western blotting.

Supplementary Material 5. Supplementary Figure S5. Knock-down of FGFR2 abrogates an increase of Nrf-2 in the nuclear fraction of the cell lysates. The expression level of Nrf-2 was determined by western blot analysis in cytoplasmic and nuclear extracts of MCF7 FGFR2(−) and T47D FGFR2(−) cells treated with FGF7 (50 ng/ml) for 4 and 8 h. Vinculin and Lamin B1 were used as loading controls for the cytoplasmic or nuclear fractions, respectively.

Supplementary Material 6. Supplementary Figure S6. FGF7/FGFR2 signalling pathway does not increase reactive oxygen species (ROS) levels in MCF7 and T47D cells. (A) Cellular ROS level was measured with flow cytometry in MCF7 and T47D cells treated with FGF7 (50 ng/ml) for 30 and 60 min, NAC (5 mM) or H_2_O_2_ (1 mM), followed by 15 min staining with H_2_-DCFDA (10 μM). Data are presented as mean fluorescence intensity (MFI) ± SD (*n* = 3), **P < 0.01, ***P < 0.001 and ns: not significant. Statistical comparisons were made using one-way ANOVA and Dunnett's multiple comparisons tests. (B) Representative histograms of ROS measured by H_2_-DCFDA.

Supplementary Material file 7. Supplementary Figure S7. Nrf-2 is involved in FGFR2-dependent response of CAMA-1 cell line to anti-ER drugs. CAMA-1 cells were cultured in 3D Matrigel® for 14 days, with FGF7 (50 ng/ml), ± 4-OHT (1 μM), ± Fulv (100 nM), ± K67 (500 nM). Representative images were taken (scale bar 100 μm), colonies were measured and analysed with ImageJ software. Quantitative data are presented as a relative ratio to CTR/non‐treated wild‐type cells, mean ± SD (*n* = 3), *P < 0.05. Statistical comparisons were made using 2-tailed Student’s *t*-test.

Supplementary Material 8. Supplementary Figure S8. FGFR2 is involved in Nrf-2-mediated response to anti-ER drugs. T47D FGFR2(−) cells were cultured in 3D Matrigel® for 14 days, with FGF7 (50 ng/ml), ± 4-OHT (1 μM), ± Fulv (100 nM), ± K67 (500 nM). Representative images were taken (scale bar 100 μm), colonies were measured and analysed with ImageJ software. Quantitative data are presented as a relative ratio to CTR/non‐treated wild‐type cells, mean ± SD (*n* = 3), ns: not significant. Statistical comparisons were made using 2-tailed Student’s *t*-test.

Supplementary Material 9. Supplementary Figure S9. Increased expression of Nrf-2 reduced sensitivity of MCF7 and T47D cells to fulvestrant. (A, B) Western blot analysis of Nrf-2 overexpression efficiency following lentiviral transduction of MCF7 and T47D cells and p62 expression level in MCF7 Nrf-2↑ and T47D Nrf-2↑ cells. (C, D) qPCR analysis of mRNA expression level of *SQSTM1* (gene encoding p62) in MCF7 Nrf-2↑ and T47D Nrf-2↑. (E, F) Proliferation of MCF7 Nrf-2↑ and T47D Nrf-2↑ cells after 72 h exposure to FGF7 (50 ng/ml) ± Fulv (0.1 μM, 0.5 μM or 1 μM) assessed by MTT assay. Quantitative data are presented as relative ratio to CTR/non‐treated wild‐type cells, mean ± SD (*n* = 3), *P < 0.05, **P < 0.01. All statistical comparisons were made using 2-tailed Student’s *t*-test.

Supplementary Material 10. Supplementary Figure S10. Knock-down of p62 re-sensitizes FGF7-treated cells to fulvestrant. (A) Western blot analysis of p62 knock-down with two different shRNA in MCF7 and T47D cells. (C, D) Proliferation of MCF7 p62(−)^1^ and T47D p62(−)^1^ cells following 72 h exposure to FGF7 (50 ng/ml) ± Fulv (0.1 μM, 0.5 μM or 1 μM) assessed by MTT assay. Quantitative data are presented as relative ratio to CTR/non‐treated wild‐type cells, mean ± SD (*n* = 3), *P < 0.05, **P < 0.01. All statistical comparisons were made using 2-tailed Student’s *t*-test.

Supplementary Material 11. Supplementary Figure S11. Distribution of age at the time of diagnosis (years) based on the level of *NFE2L2* gene expression (*NFE2L2*-low and *NFE2L2*-high) within *FGFR2*-high subgroup of patients (*n* = 550). The analysis was performed using the Mann-Whitney-Wilcoxon test

Supplementary Material 12. Supplementary Figure S12. Images of uncropped Western blot membranes.

Additional file13. Supplementary Table 1-3.

## Data Availability

The datasets used and/or analysed during the current study are available from the corresponding author on reasonable request.

## Abbreviations

BCa: Breast cancer
FGFR2: Fibroblast growth factor receptor 2
OHT: 4-Hydroxytamoxifen
Fulv: Fulvestrant
CQ: Chloroquine
PLA: Proximity ligation assay
ROS: Reactive oxygen species
tBHQ: *tert*-Butylhydroquinone
