# Supplementary figures and images for: FGFR2-triggered autophagy and activation of Nrf-2 reduce breast cancer cell response to anti-ER drugs
Source: Cell Mol Biol Lett. 2024 May 14;29:71. doi: 10.1186/s11658-024-00586-6 (PMC11092031; doi:10.1186/s11658-024-00586-6)

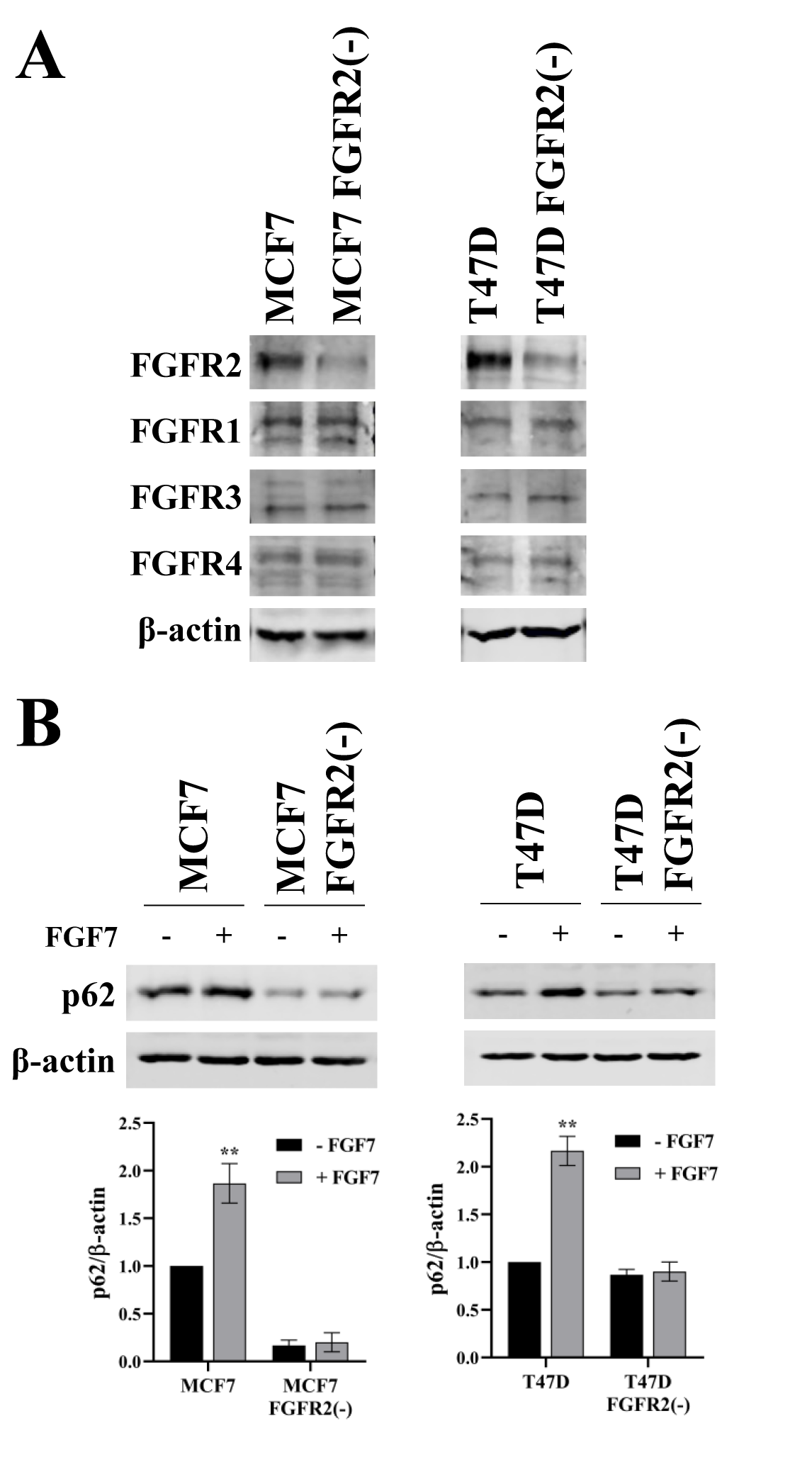

Supplement: Supplementary file 1 — Supplementary Material 1. Supplementary Figure S1. FGFR2 mediates FGF7-dependent effect on the expression of p62. (A) Knock-down of FGFR2 and the expression levels of FGFR1, FGFR3 and FGFR4 were verified by western blotting in MCF7 and T47D cells. (B) Evaluation of p62 expression level by western blotting in MCF7, MCF7 FGFR2(−), T47D and T47D FGFR2(−) cells treated with FGF7 (50 ng/ml) for 24 h. All quantitative data are presented as the mean ± SD (n = 3), **P < 0.01. Statistical comparisons were made using 2-tailed Student’s t-test. [file 11658_2024_586_MOESM1_ESM.tif]

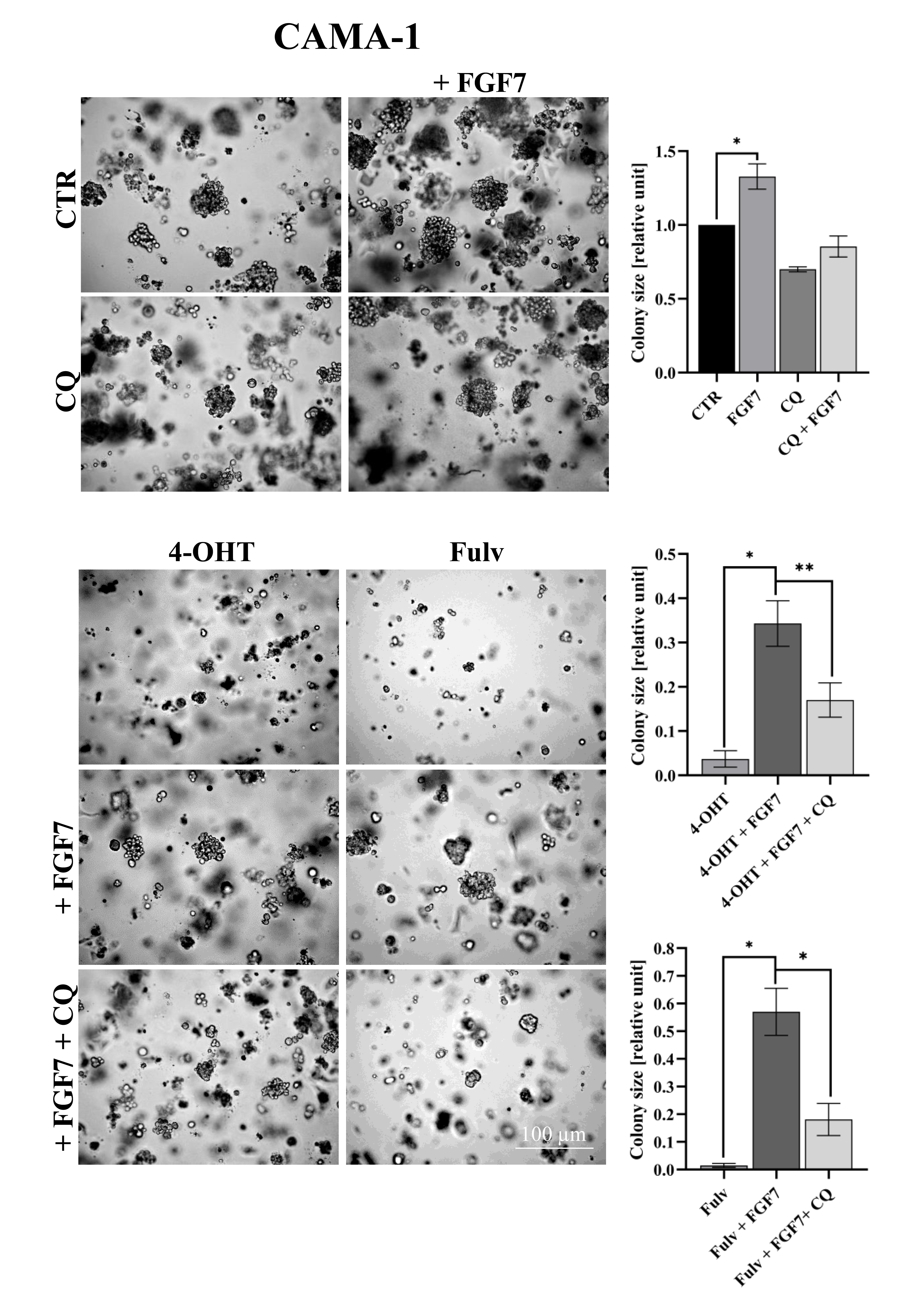

Supplement: Supplementary file 2 — Supplementary Material 2. Supplementary Figure S3. Inhibition of autophagy restores sensitivity to anti-ER drugs in CAMA-1 cell line. CAMA-1 cells were grown in 3D Matrigel® for 14 day in the presence of FGF7 (50 ng/ml), ± 4-OHT (1 μM), ± Fulv (100 nM), ± CQ (1 μM). Representative images were taken (scale bar 100 μm), colonies were measured and analysed with ImageJ software. All quantitative data are presented as a relative ratio to CTR/non‐treated wild‐type cells, mean ± SD (n = 3), *P < 0.05 and **P < 0.01. Statistical comparisons were made using 2-tailed Student’s t-test. [file 11658_2024_586_MOESM2_ESM.tif]

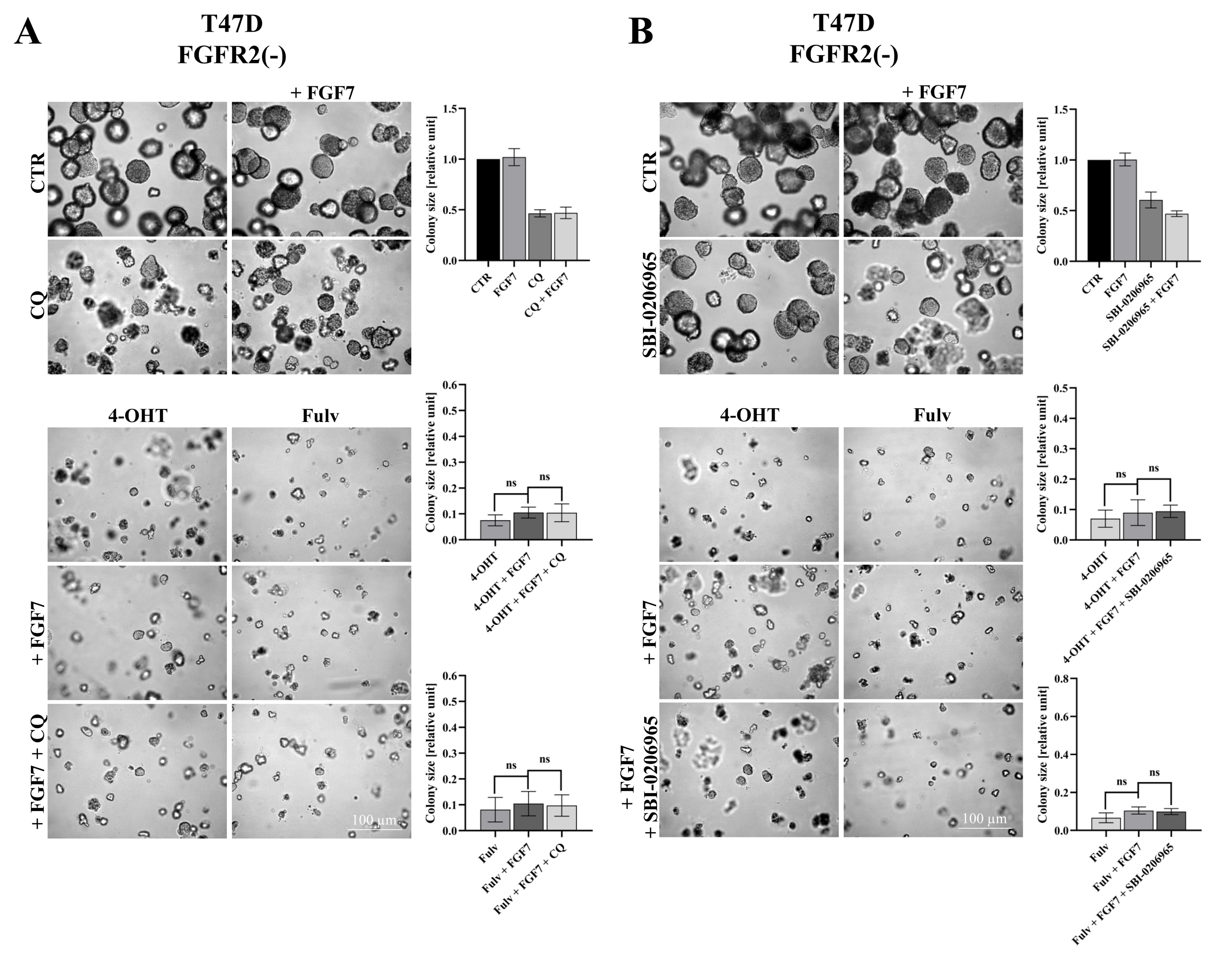

Supplement: Supplementary file 3 — Supplementary Material 3. Supplementary Figure S3. Knock-down of FGFR2 abolishes the effects of FGF7-induced autophagy. (A) T47D FGFR2(−) cells were cultured in 3D Matrigel® for 14 days with FGF7 (50 ng/ml), ± 4-OHT (1 μM), ± Fulv (100 nM), ± CQ (1 μM). (C) T47D FGFR2(−) cells were cultured in 3D Matrigel® for 14 days with FGF7 (50 ng/ml) ± 4-OHT (1 μM), ± Fulv (100 nM), ± SBI-0206965 (750 nM). Representative images were taken (scale bar 100 μm), colonies were measured and analysed with ImageJ software. All quantitative data are presented as a relative ratio to CTR/non‐treated wild‐type cells, mean ± SD (n = 3), ns: not significant. All statistical comparisons were made using 2-tailed Student’s t-test. [file 11658_2024_586_MOESM3_ESM.tif]

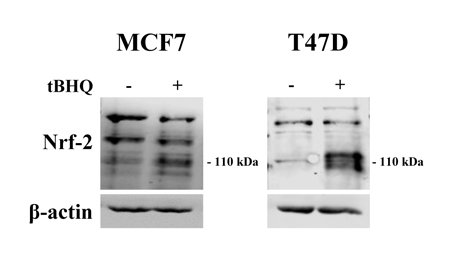

Supplement: Supplementary file 4 — Supplementary Material 4. Supplementary Figure S4. Induction of Nrf-2 expression by tBHQ. To establish the specificity of anti-Nrf-2 antibody, MCF7 and T47D cells were treated for 24 h with tBHQ (50 μM). Nrf-2 expression was analysed by western blotting. [file 11658_2024_586_MOESM4_ESM.tif]

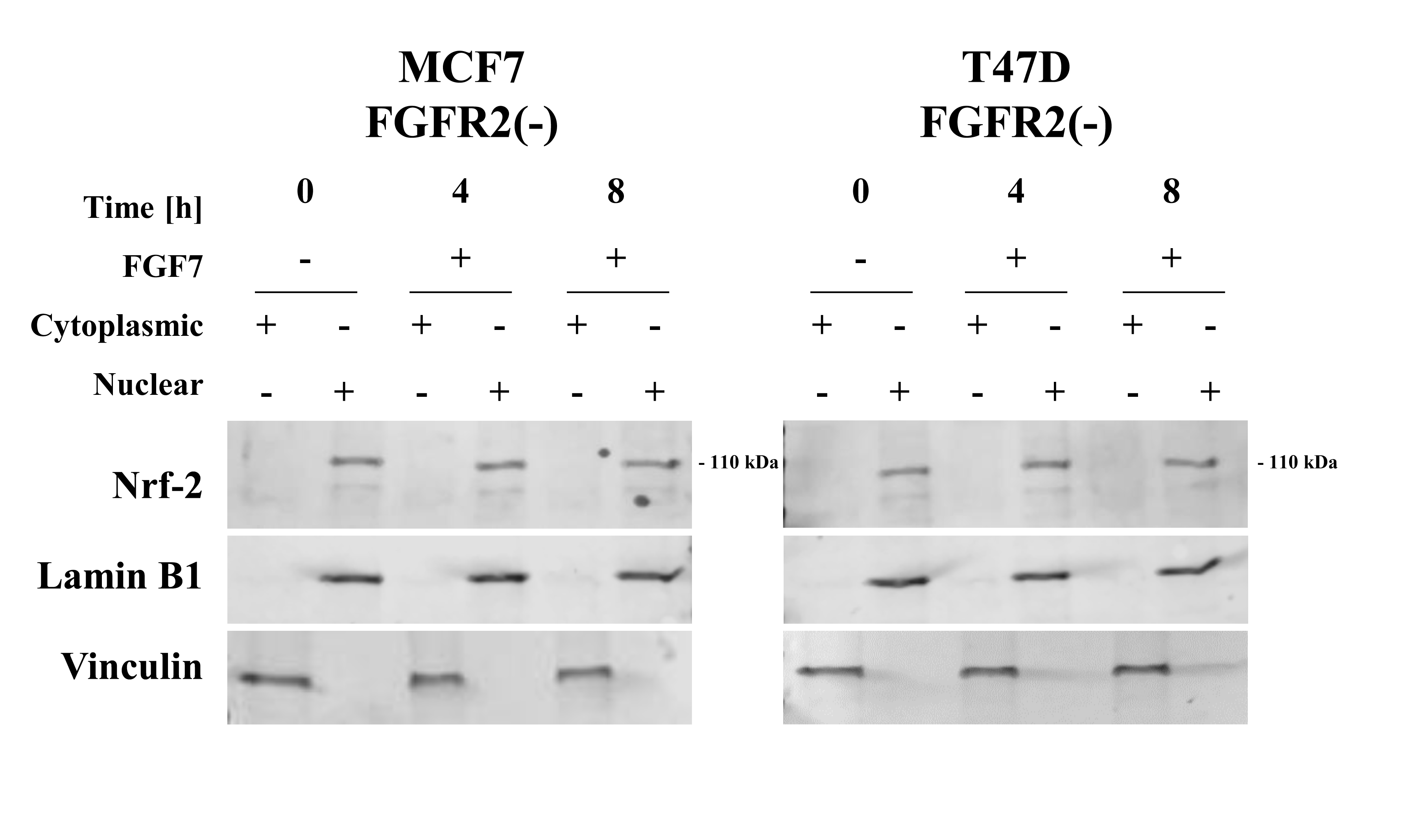

Supplement: Supplementary file 5 — Supplementary Material 5. Supplementary Figure S5. Knock-down of FGFR2 abrogates an increase of Nrf-2 in the nuclear fraction of the cell lysates. The expression level of Nrf-2 was determined by western blot analysis in cytoplasmic and nuclear extracts of MCF7 FGFR2(−) and T47D FGFR2(−) cells treated with FGF7 (50 ng/ml) for 4 and 8 h. Vinculin and Lamin B1 were used as loading controls for the cytoplasmic or nuclear fractions, respectively. [file 11658_2024_586_MOESM5_ESM.tif]

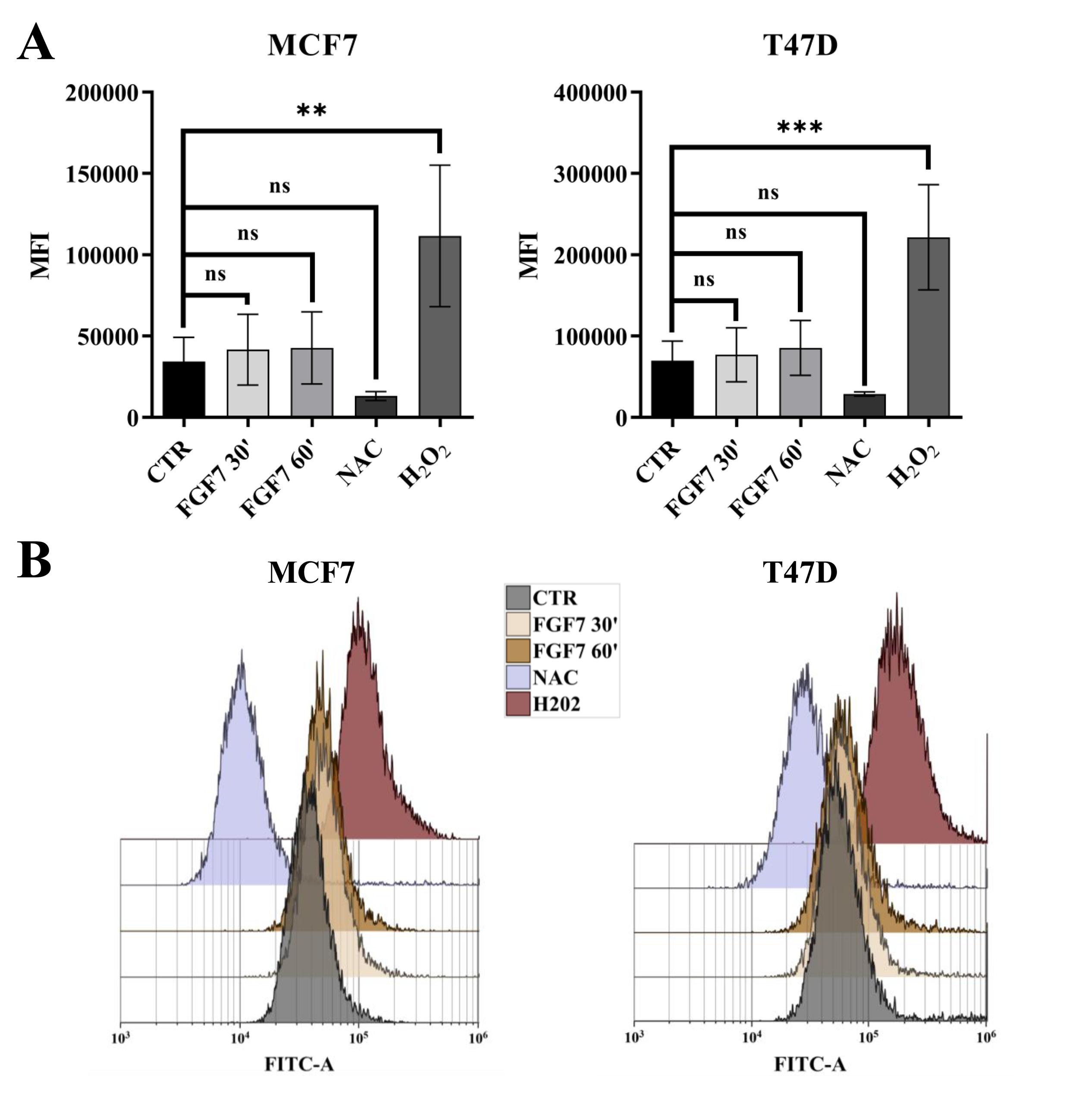

Supplement: Supplementary file 6 — Supplementary Material 6. Supplementary Figure S6. FGF7/FGFR2 signalling pathway does not increase reactive oxygen species (ROS) levels in MCF7 and T47D cells. (A) Cellular ROS level was measured with flow cytometry in MCF7 and T47D cells treated with FGF7 (50 ng/ml) for 30 and 60 min, NAC (5 mM) or H2O2 (1 mM), followed by 15 min staining with H2-DCFDA (10 μM). Data are presented as mean fluorescence intensity (MFI) ± SD (n = 3), **P < 0.01, ***P < 0.001 and ns: not significant. Statistical comparisons were made using one-way ANOVA and Dunnett's multiple comparisons tests. (B) Representative histograms of ROS measured by H2-DCFDA. [file 11658_2024_586_MOESM6_ESM.tif]

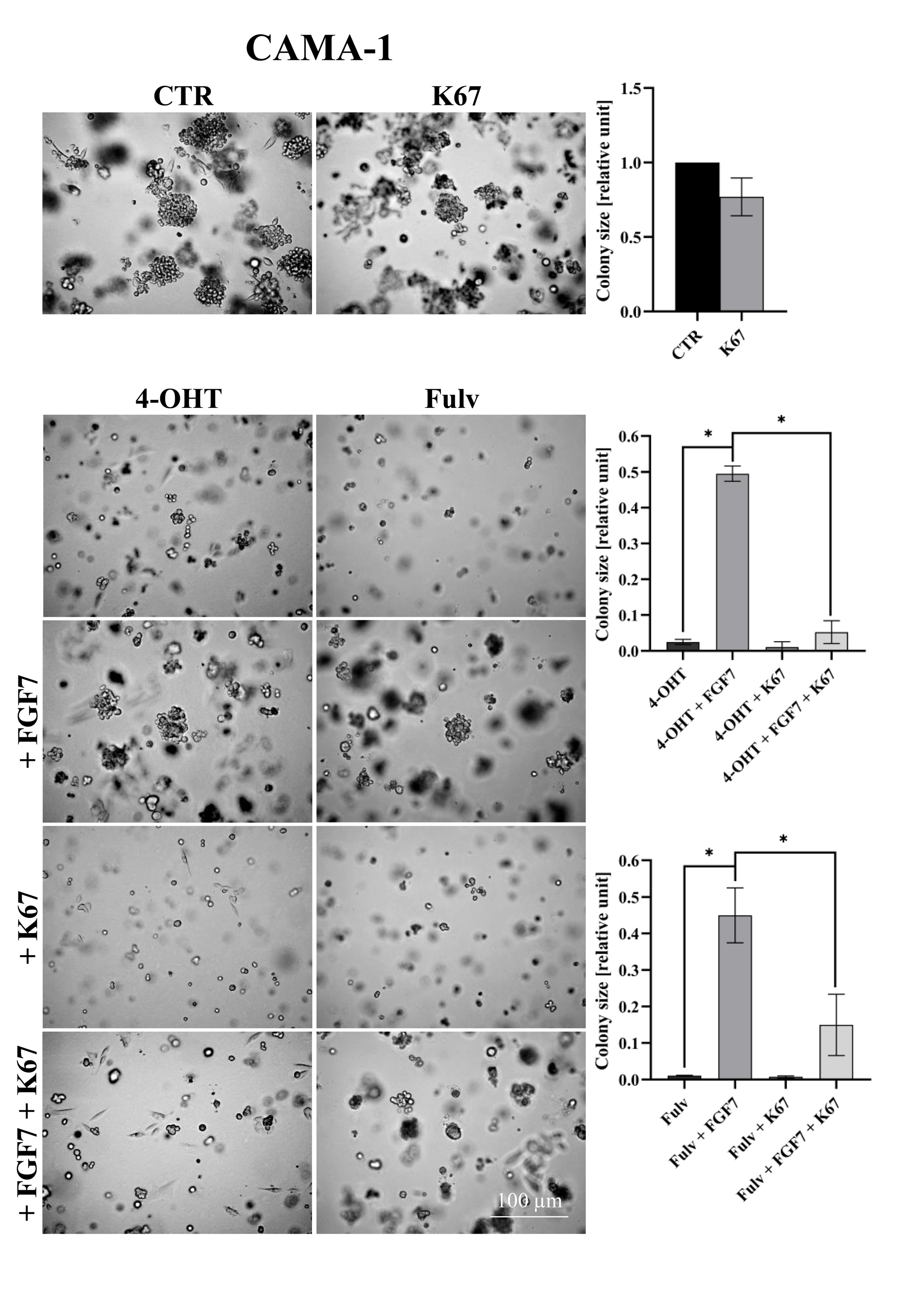

Supplement: Supplementary file 7 — Supplementary Material file 7. Supplementary Figure S7. Nrf-2 is involved in FGFR2-dependent response of CAMA-1 cell line to anti-ER drugs. CAMA-1 cells were cultured in 3D Matrigel® for 14 days, with FGF7 (50 ng/ml), ± 4-OHT (1 μM), ± Fulv (100 nM), ± K67 (500 nM). Representative images were taken (scale bar 100 μm), colonies were measured and analysed with ImageJ software. Quantitative data are presented as a relative ratio to CTR/non‐treated wild‐type cells, mean ± SD (n = 3), *P < 0.05. Statistical comparisons were made using 2-tailed Student’s t-test. [file 11658_2024_586_MOESM7_ESM.tif]

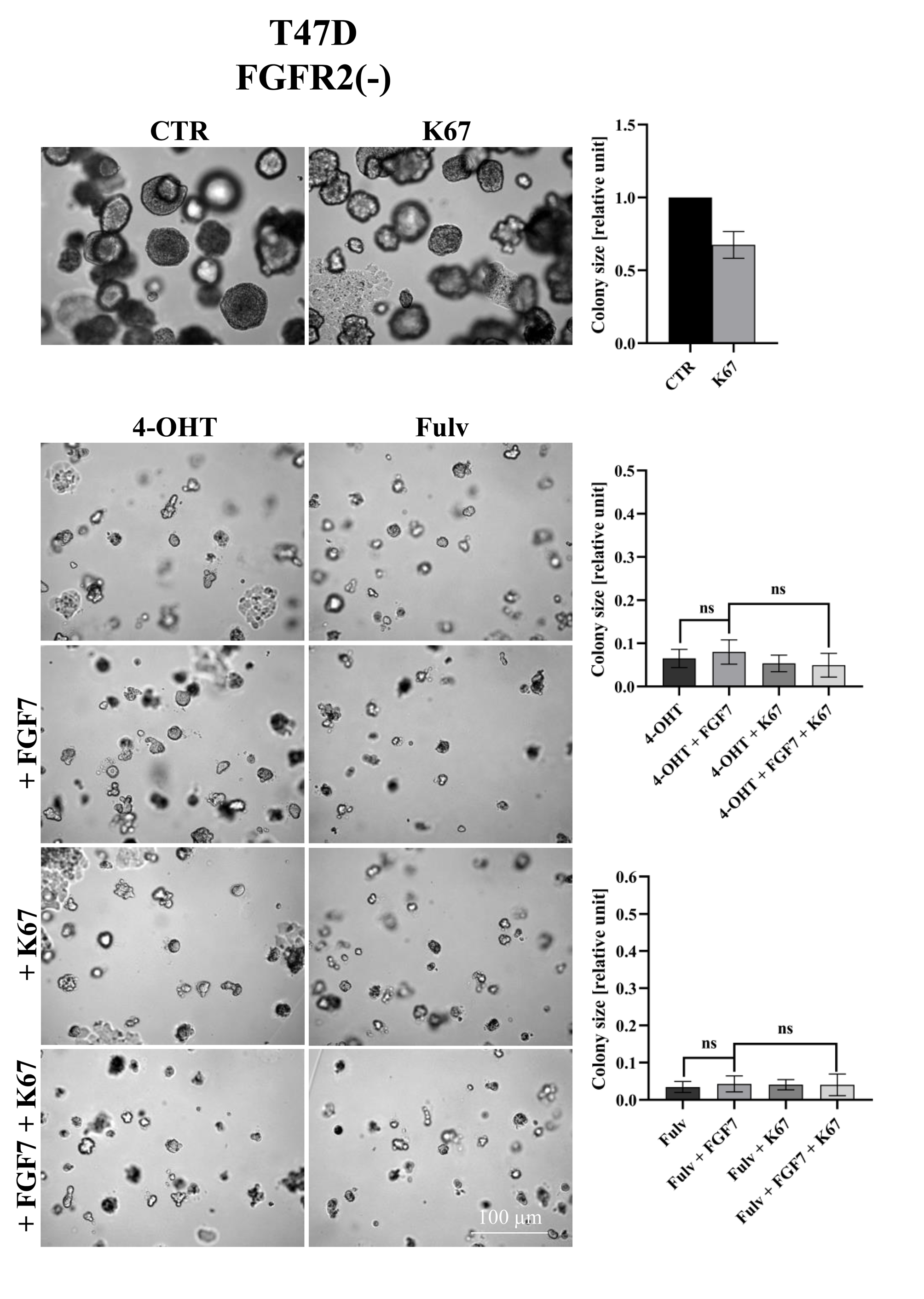

Supplement: Supplementary file 8 — Supplementary Material 8. Supplementary Figure S8. FGFR2 is involved in Nrf-2-mediated response to anti-ER drugs. T47D FGFR2(−) cells were cultured in 3D Matrigel® for 14 days, with FGF7 (50 ng/ml), ± 4-OHT (1 μM), ± Fulv (100 nM), ± K67 (500 nM). Representative images were taken (scale bar 100 μm), colonies were measured and analysed with ImageJ software. Quantitative data are presented as a relative ratio to CTR/non‐treated wild‐type cells, mean ± SD (n = 3), ns: not significant. Statistical comparisons were made using 2-tailed Student’s t-test. [file 11658_2024_586_MOESM8_ESM.tif]

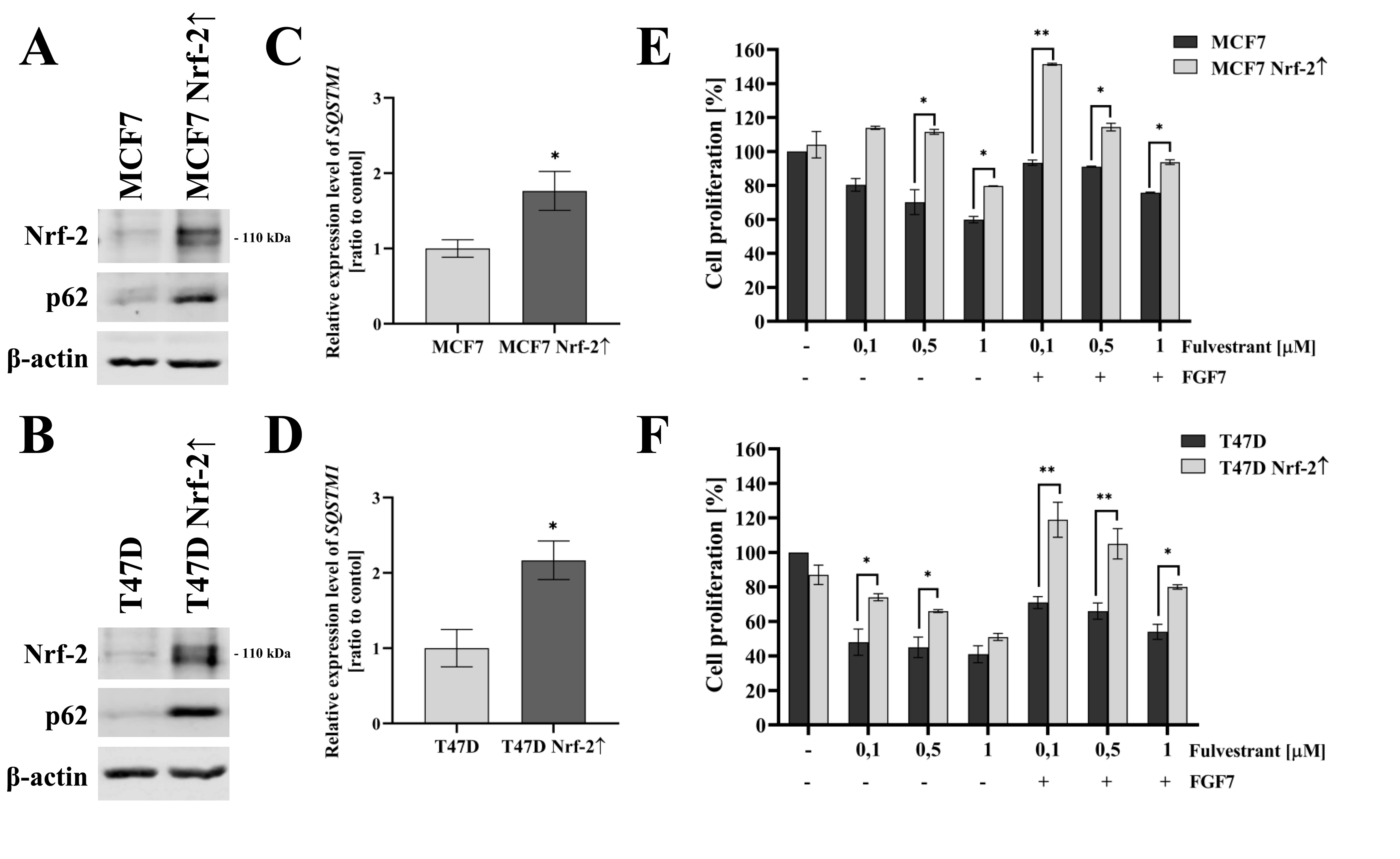

Supplement: Supplementary file 9 — Supplementary Material 9. Supplementary Figure S9. Increased expression of Nrf-2 reduced sensitivity of MCF7 and T47D cells to fulvestrant. (A, B) Western blot analysis of Nrf-2 overexpression efficiency following lentiviral transduction of MCF7 and T47D cells and p62 expression level in MCF7 Nrf-2↑ and T47D Nrf-2↑ cells. (C, D) qPCR analysis of mRNA expression level of SQSTM1 (gene encoding p62) in MCF7 Nrf-2↑ and T47D Nrf-2↑. (E, F) Proliferation of MCF7 Nrf-2↑ and T47D Nrf-2↑ cells after 72 h exposure to FGF7 (50 ng/ml) ± Fulv (0.1 μM, 0.5 μM or 1 μM) assessed by MTT assay. Quantitative data are presented as relative ratio to CTR/non‐treated wild‐type cells, mean ± SD (n = 3), *P < 0.05, **P < 0.01. All statistical comparisons were made using 2-tailed Student’s t-test. [file 11658_2024_586_MOESM9_ESM.tif]

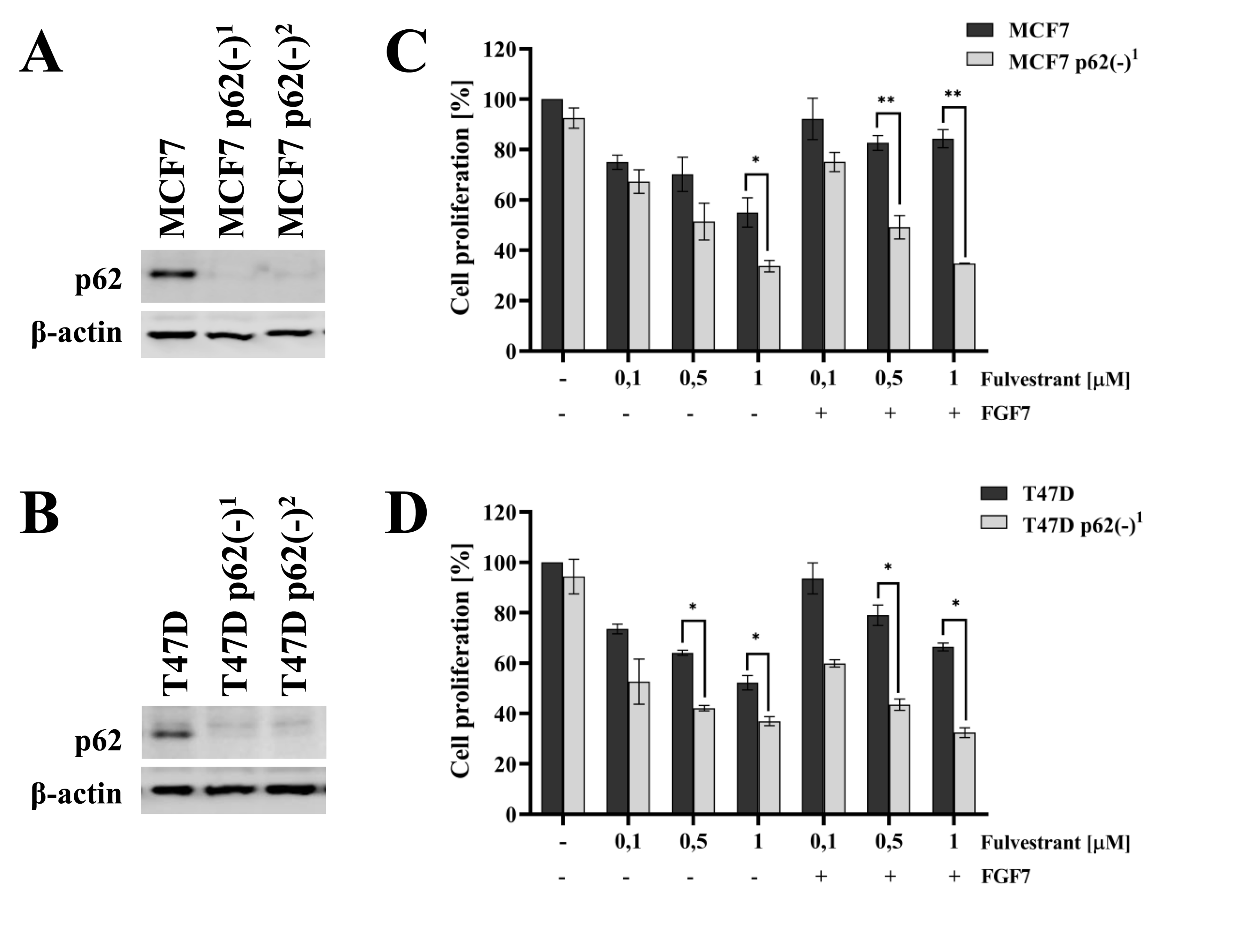

Supplement: Supplementary file 10 — Supplementary Material 10. Supplementary Figure S10. Knock-down of p62 re-sensitizes FGF7-treated cells to fulvestrant. (A) Western blot analysis of p62 knock-down with two different shRNA in MCF7 and T47D cells. (C, D) Proliferation of MCF7 p62(−)1 and T47D p62(−)1 cells following 72 h exposure to FGF7 (50 ng/ml) ± Fulv (0.1 μM, 0.5 μM or 1 μM) assessed by MTT assay. Quantitative data are presented as relative ratio to CTR/non‐treated wild‐type cells, mean ± SD (n = 3), *P < 0.05, **P < 0.01. All statistical comparisons were made using 2-tailed Student’s t-test. [file 11658_2024_586_MOESM10_ESM.tif]

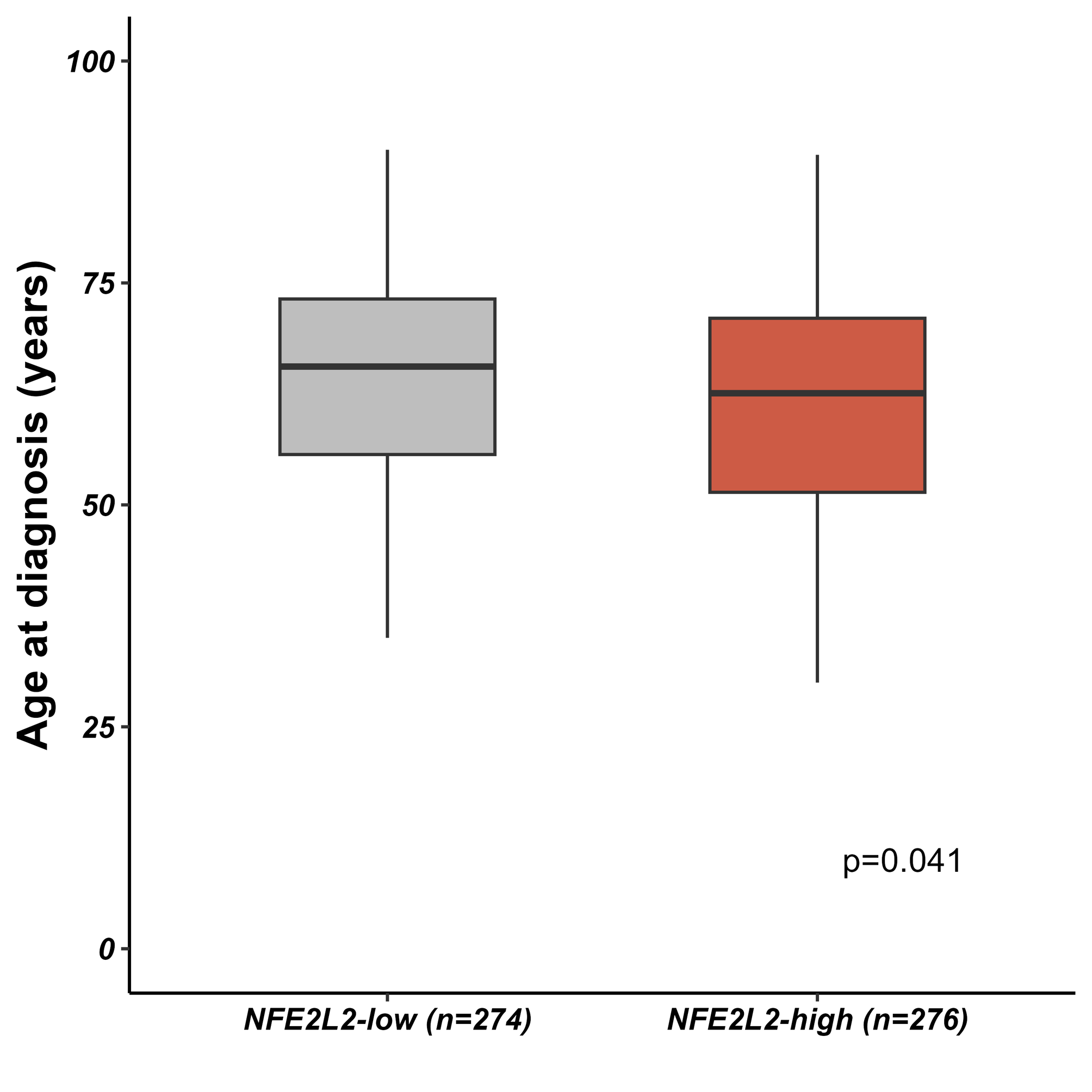

Supplement: Supplementary file 11 — Supplementary Material 11. Supplementary Figure S11. Distribution of age at the time of diagnosis (years) based on the level of NFE2L2 gene expression (NFE2L2-low and NFE2L2-high) within FGFR2-high subgroup of patients (n = 550). The analysis was performed using the Mann-Whitney-Wilcoxon test [file 11658_2024_586_MOESM11_ESM.tif]

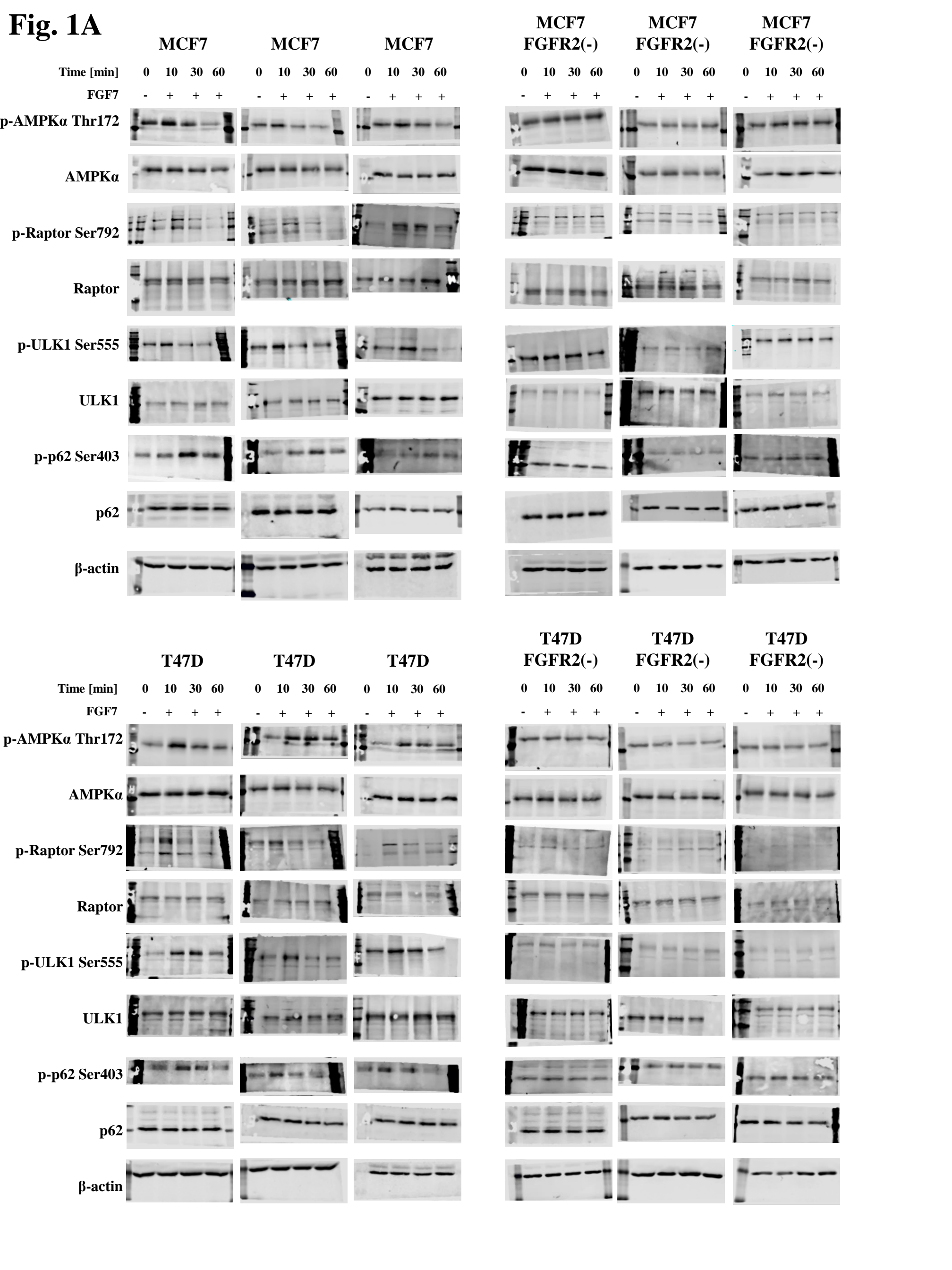

**Fig. 1C**

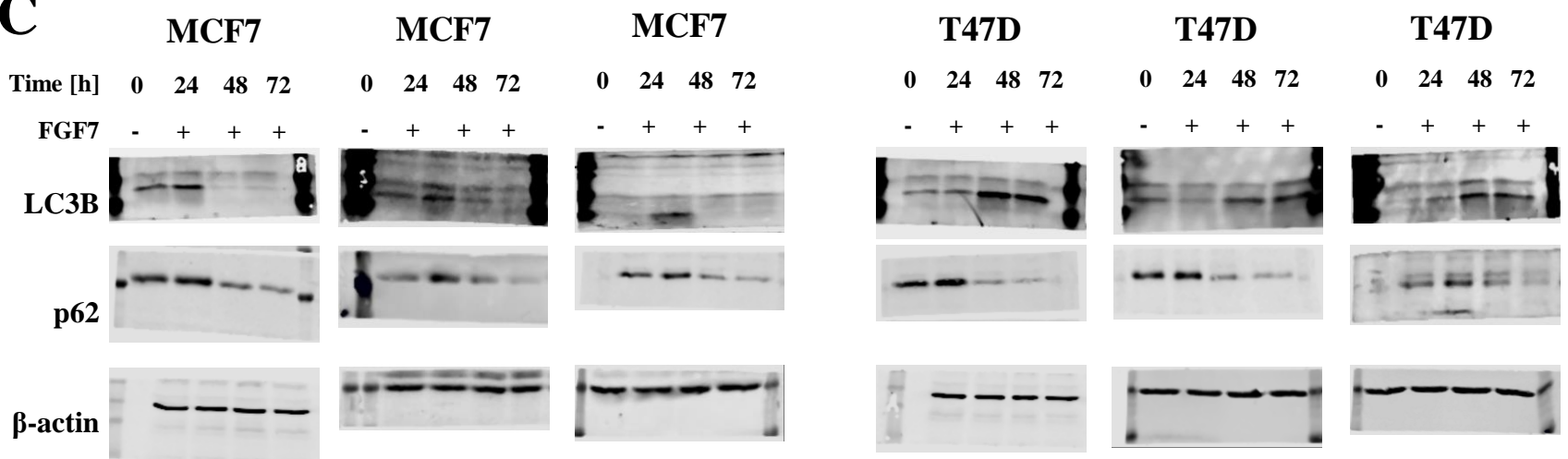

**Fig. 1D**

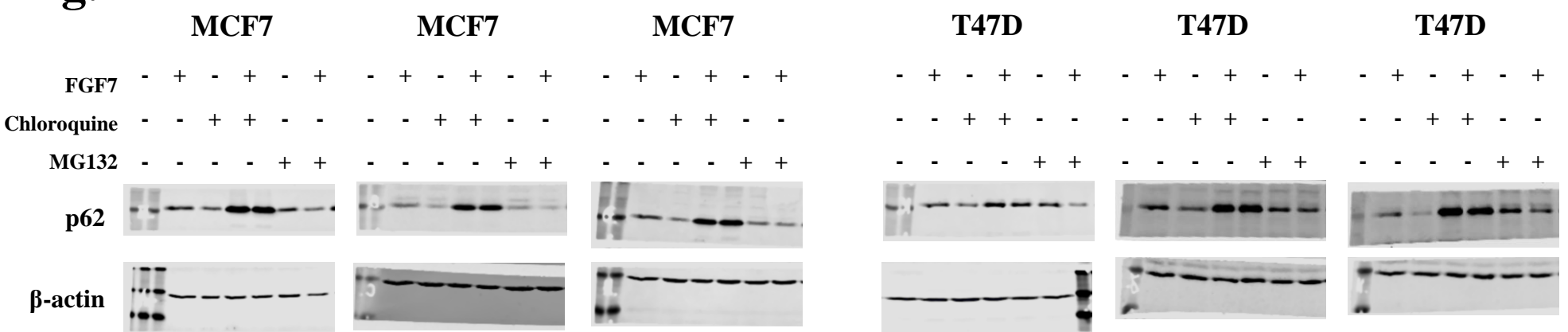

**Fig. 3A**

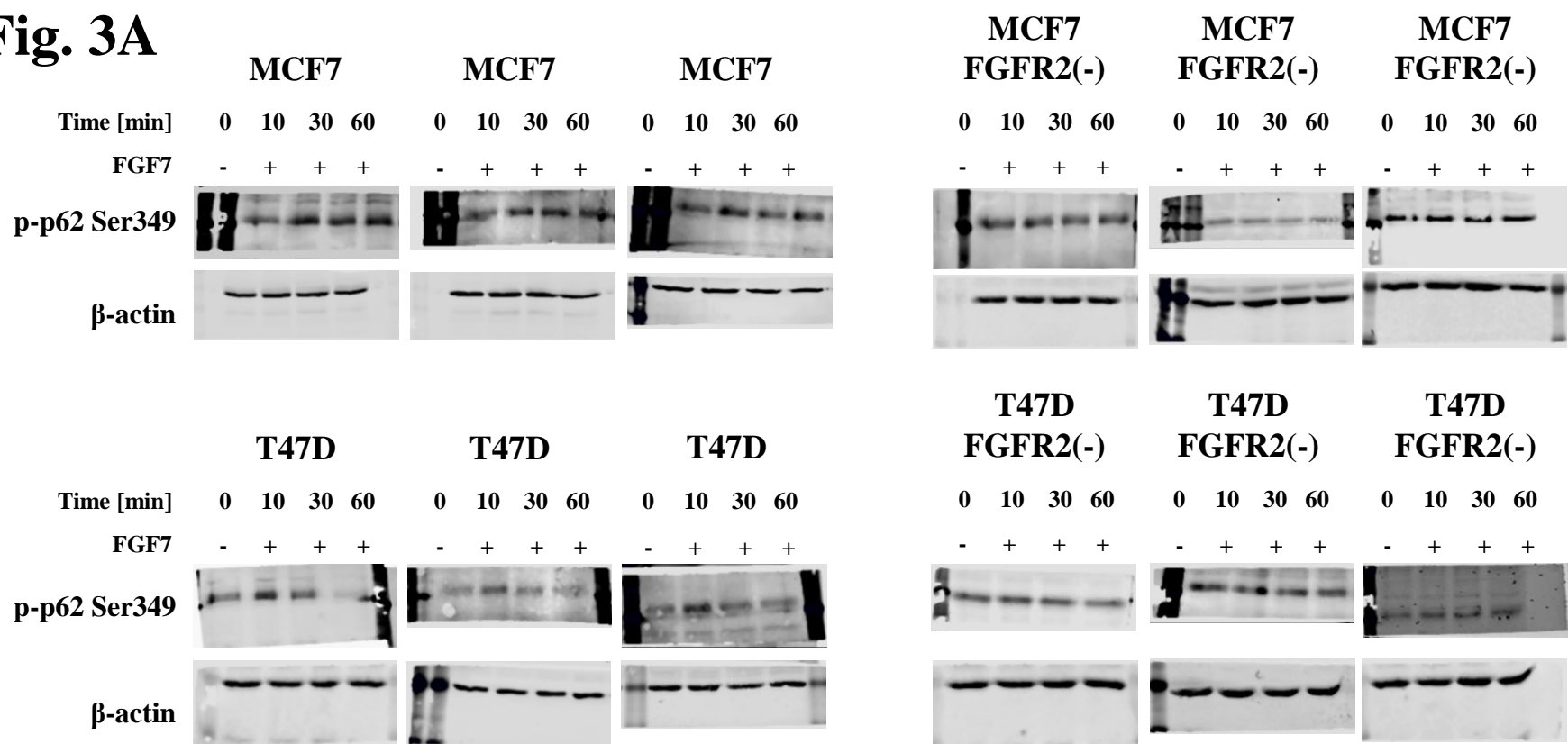

**Fig. 3B**

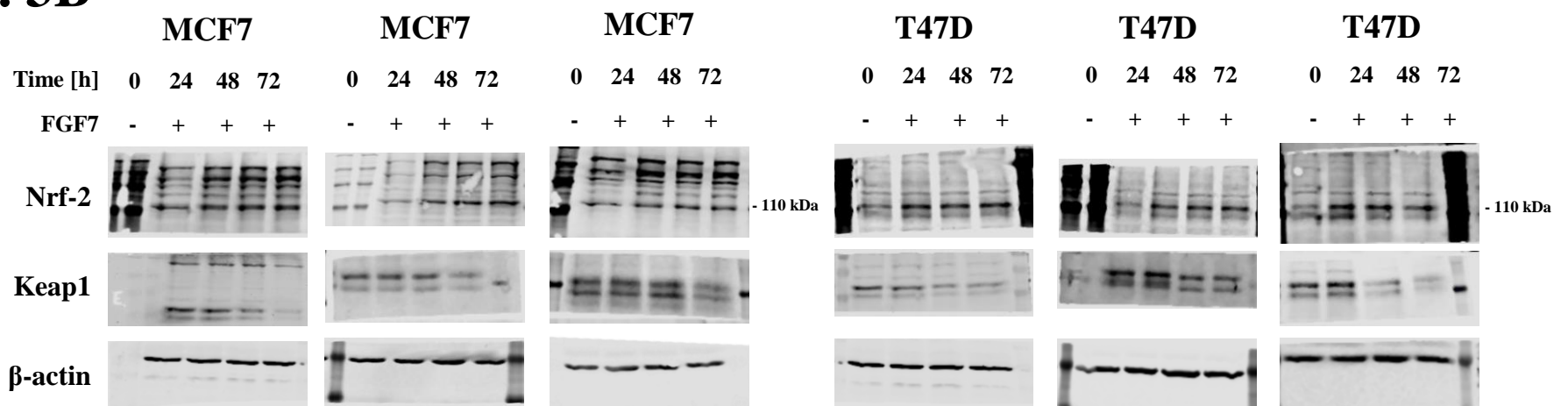

Fig. 3B cont.

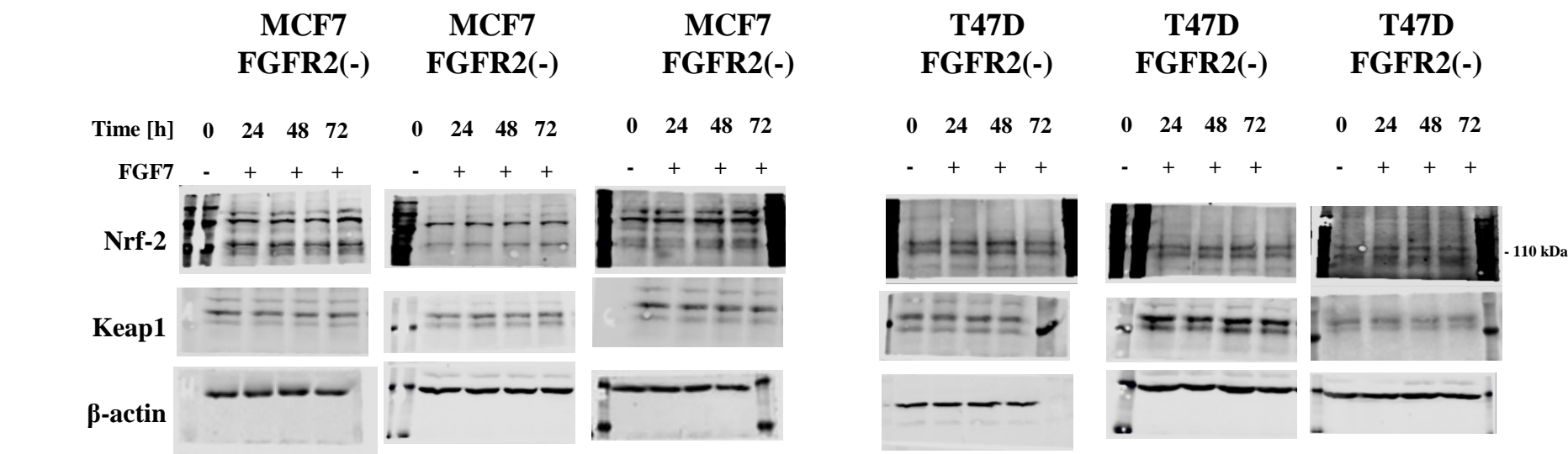

Fig. 3C

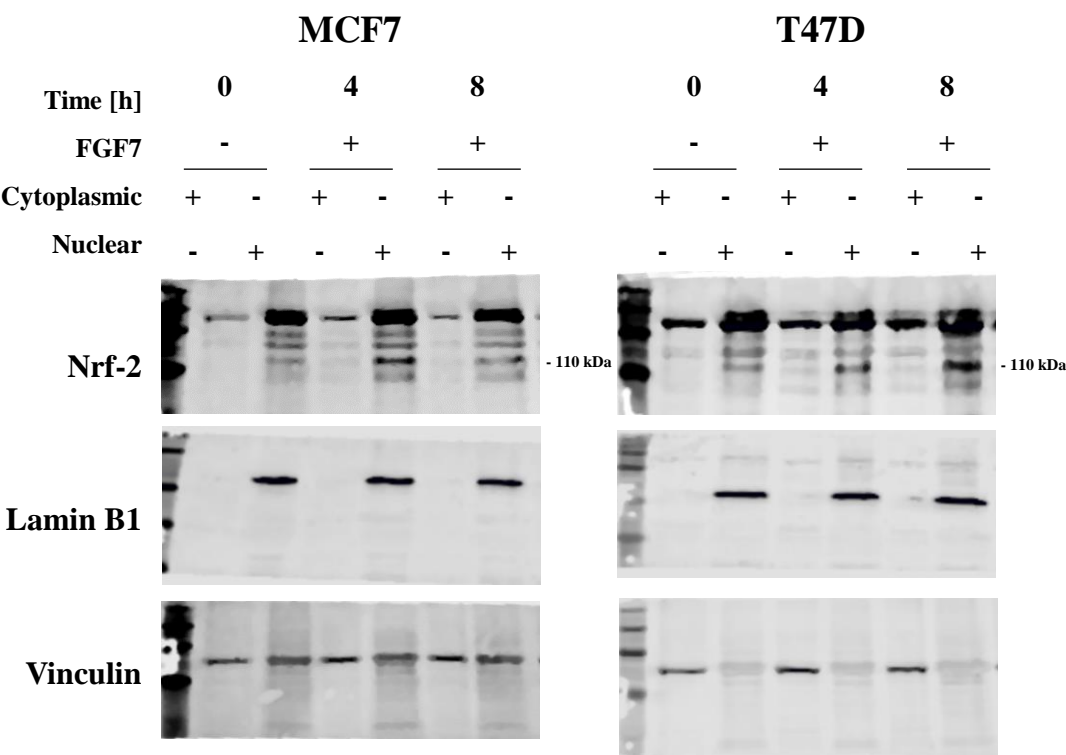

Fig. S1A

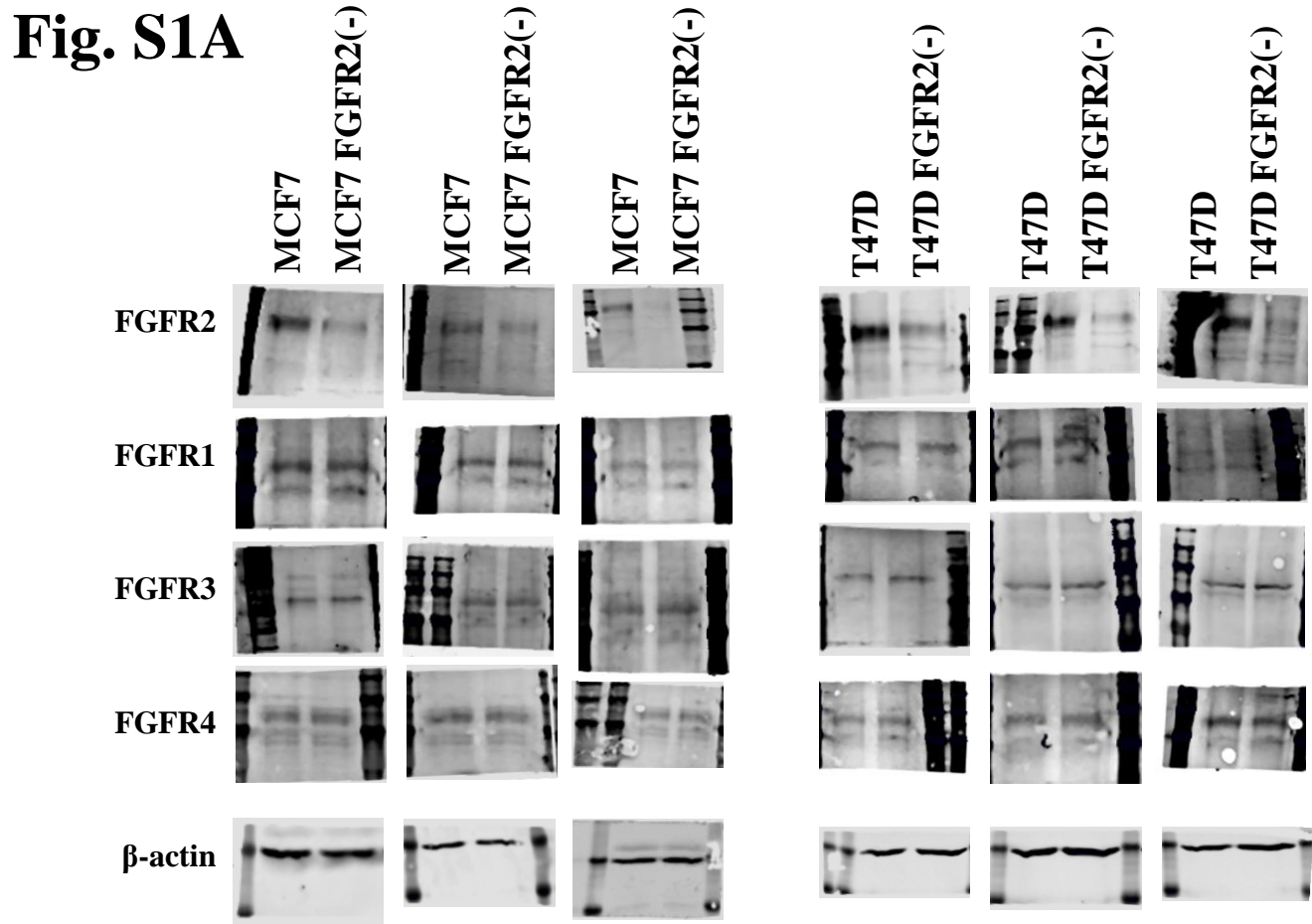

Fig. S1B

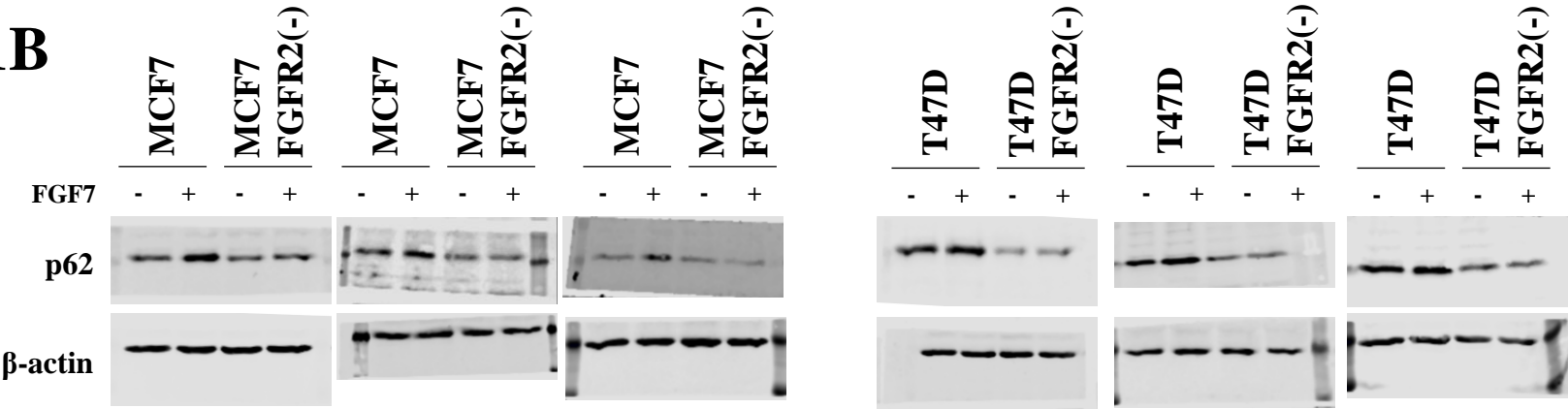

Fig. S4

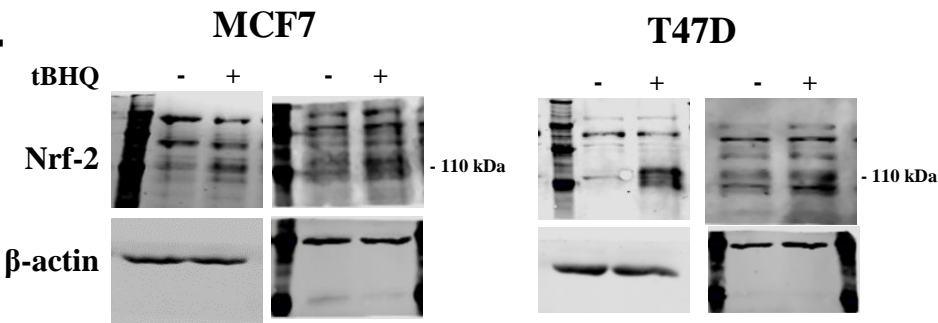

Fig. S5

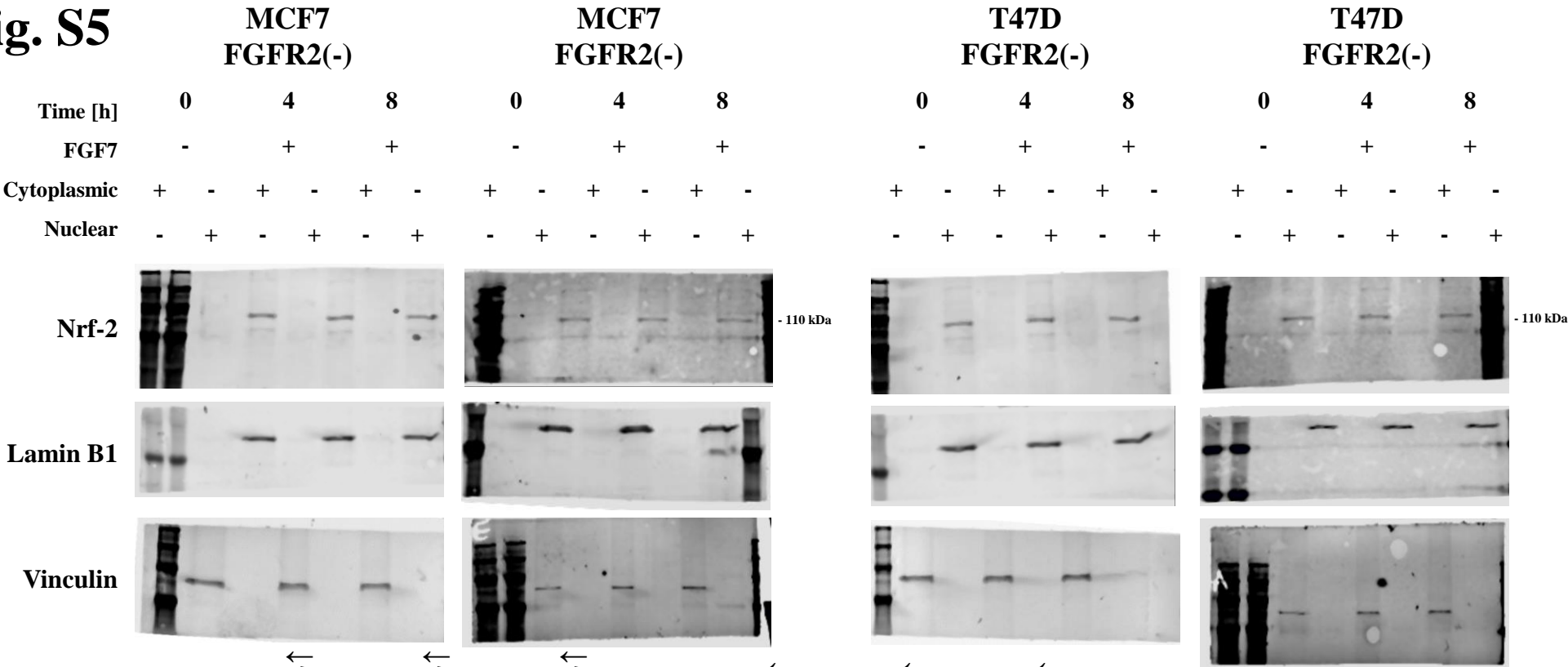

Fig. S9A-B

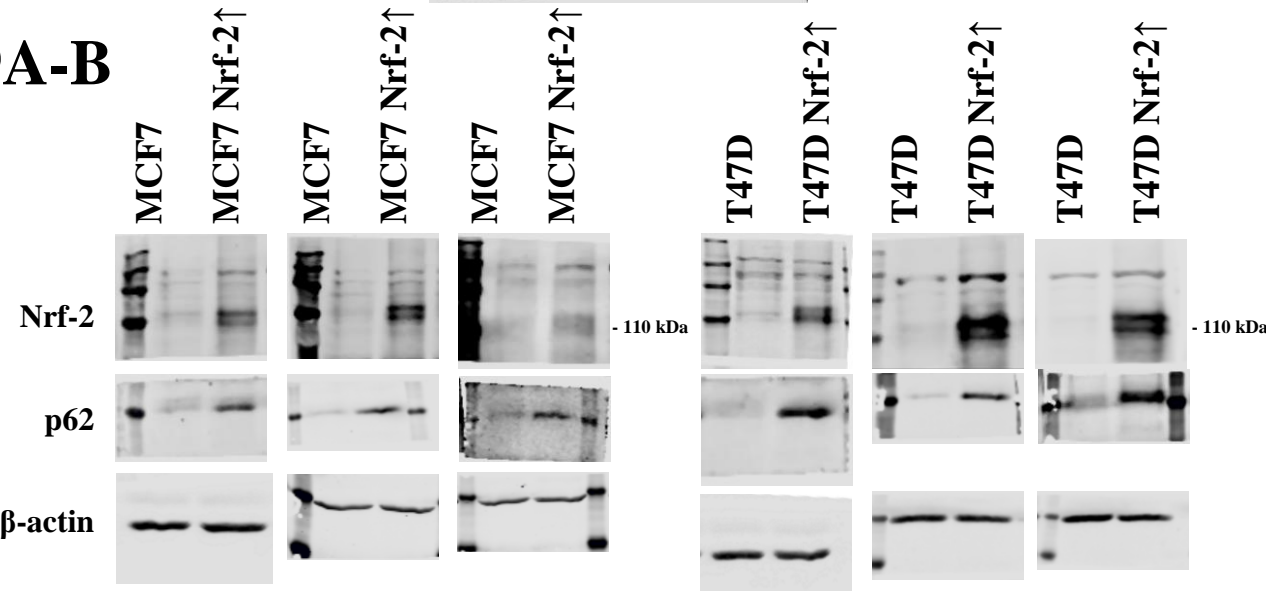

Fig. S10A-B

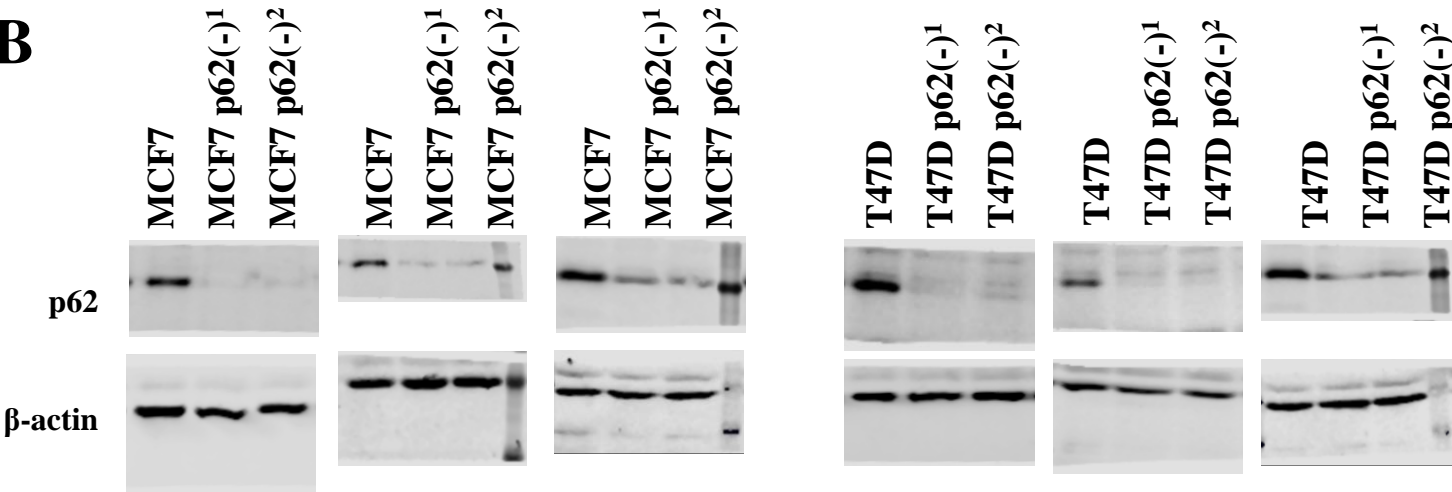

Supplement: Supplementary file 12 — Supplementary Material 12. Supplementary Figure S12. Images of uncropped Western blot membranes. [file 11658_2024_586_MOESM12_ESM.pdf]
