# Supplementary material for: FGFR2-triggered autophagy and activation of Nrf-2 reduce breast cancer cell response to anti-ER drugs
Source: Cell Mol Biol Lett. 2024 May 14;29:71. doi: 10.1186/s11658-024-00586-6 (PMC11092031; doi:10.1186/s11658-024-00586-6)
Supplement: Supplementary file 13 — Additional file13. Supplementary Table 1-3. [file 11658_2024_586_MOESM13_ESM.docx]

Supplementary Table 1. Pathological and clinical characteristics of the *FGFR2*-high group (n = 550).

| **Variable** |  | **median** | **IQR** |
| --- | --- | --- | --- |
| Age at diagnosis [years] |  | 64 | 53-72 |
| Tumor size [mm] |  | 21 | 17-30 |
| **Variable** |  | **n** | **%** |
| Histological subtype | NST | 455 | 83 |
|  | mixed | 95 | 17 |
| Grade | G1 | 71 | 13 |
|  | G2 | 269 | 51 |
|  | G3 | 189 | 36 |
|  | NA | 21 | - |
| Tumor stage | I | 155 | 38 |
|  | II | 240 | 58 |
|  | III | 16 | 4 |
|  | IV | 2 | 0 |
|  | NA | 137 | - |
| Menopausal status | pre | 102 | 19 |
|  | post | 448 | 81 |
| ER status | negative | 0 | 0 |
|  | positive | 550 | 100 |
| PR status | negative | 0 | 0 |
|  | positive | 550 | 100 |
| HER2 status | negative | 524 | 95 |
|  | positive | 26 | 5 |
| Chemotherapy | no | 497 | 90 |
|  | yes | 53 | 10 |
| Hormonotherapy | no | 157 | 29 |
|  | yes | 393 | 71 |
| Radiotherapy | no | 228 | 41 |
|  | yes | 322 | 59 |

Nominal variables are presented as raw values followed by percentages, continuous variables are presented as medians and interquartile ranges. Gene expression data and clinical characteristics of patients from METABRIC cohort were obtained from cBioPortal™ for Cancer Genomics (Breast Cancer, METABRIC dataset).

Supplementary Table 2. Pathological and clinical characteristics of the *FGFR2*-high subgroup of patients according to *NFE2L2* mRNA level.

|  |  | **NFE2L2-low (n=274)** | | **NFE2L2-high (n=276)** | | **Mann-Whitney-Wilcoxon test** |
| --- | --- | --- | --- | --- | --- | --- |
| **Variable** |  | **median** | **IQR** | **median** | **IQR** | **p-value** |
| Age at diagnosis [years] | | 66 | 56-73 | 63 | 51-71 | 0.041 |
| Tumor size [mm] | | 22 | 18-30 | 21 | 16-27 | 0.185 |
|  |  |  |  |  |  |  |
|  | | **NFE2L2-low (n=274)** | | **NFE2L2-high (n=276)** | | **Fisher's Exact Test** |
| **Variable** | | **n** | **%** | **n** | **%** | **p-value** |
| Histological subtype | NST | 233 | 84 | 225 | 82 | 0.499 |
|  | mixed | 44 | 16 | 51 | 18 |  |
| Grade | G1 | 30 | 12 | 41 | 15 | 0.326 |
|  | G2 | 129 | 50 | 140 | 52 |  |
|  | G3 | 99 | 38 | 90 | 33 |  |
|  | NA | 16 | - | 5 | - |  |
| Tumor stage | I | 73 | 37 | 82 | 38 | 0.904 |
|  | II | 114 | 58 | 126 | 58 |  |
|  | III | 9 | 5 | 7 | 3 |  |
|  | IV | 1 | 1 | 1 | 0 |  |
|  | NA | 77 | - | 60 | - |  |
| HER2 status | negative | 259 | 95 | 265 | 96 | 0.430 |
|  | positive | 15 | 5 | 11 | 4 |  |
| Chemotherapy | no | 252 | 92 | 245 | 89 | 0.248 |
|  | yes | 22 | 8 | 31 | 11 |  |
| Hormonotherapy | no | 93 | 34 | 64 | 23 | 0.006 |
|  | yes | 181 | 66 | 212 | 77 |  |
| Radiotherapy | no | 112 | 41 | 116 | 42 | 0.796 |
|  | yes | 162 | 59 | 160 | 58 |  |

Nominal variables are presented as raw values followed by percentages of the respective groups, continuous variables are presented as medians and interquartile ranges. Gene expression data and clinical characteristics of patients from METABRIC cohort were obtained from cBioPortal™ for Cancer Genomics (Breast Cancer, METABRIC dataset).

Supplementary Table 3. Distribution of the expression level of the *NFE2L2* and *FGFR2* genes within the studied group.

|  | ***FGFR2*-low** | ***FGFR2*-high** |
| --- | --- | --- |
| ***NFE2L2*-low** | 17% (139) | 33% (274) |
| ***NFE2L2*-high** | 16% (136) | 33% (276) |

The expression level of the *NFE2L2* was dichotomized by median, whereas *FGFR2* was dichotomized by 1st tercile. Data presented as percentages and numbers in brackets. Gene expression data and clinical characteristics of patients from METABRIC cohort were obtained from cBioPortal™ for Cancer Genomics (Breast Cancer, METABRIC dataset).
